# Revisiting the outcome of adult wild-type *Htt* inactivation in the context of *HTT*-lowering strategies for Huntington’s disease

**DOI:** 10.1093/braincomms/fcad344

**Published:** 2023-12-07

**Authors:** Sara Regio, Gabriel Vachey, Enrique Goñi, Fabio Duarte, Margareta Rybarikova, Mélanie Sipion, Maria Rey, Maite Huarte, Nicole Déglon

**Affiliations:** Lausanne University Hospital (CHUV) and University of Lausanne (UNIL), Department of Clinical Neurosciences (DNC), Laboratory of Cellular and Molecular Neurotherapies (LCMN), Lausanne 1011, Switzerland; Lausanne University Hospital (CHUV) and University of Lausanne (UNIL), Neuroscience Research Center (CRN), Laboratory of Cellular and Molecular Neurotherapies (LCMN), Lausanne 1011, Switzerland; Lausanne University Hospital (CHUV) and University of Lausanne (UNIL), Department of Clinical Neurosciences (DNC), Laboratory of Cellular and Molecular Neurotherapies (LCMN), Lausanne 1011, Switzerland; Lausanne University Hospital (CHUV) and University of Lausanne (UNIL), Neuroscience Research Center (CRN), Laboratory of Cellular and Molecular Neurotherapies (LCMN), Lausanne 1011, Switzerland; Center for Applied Medical Research, University of Navarra, Pamplona 31008, Spain; Institute of Health Research of Navarra (IdiSNA), Cancer Center Clínica Universidad de Navarra (CCUN), Pamplona 31008, Spain; Lausanne University Hospital (CHUV) and University of Lausanne (UNIL), Department of Clinical Neurosciences (DNC), Laboratory of Cellular and Molecular Neurotherapies (LCMN), Lausanne 1011, Switzerland; Lausanne University Hospital (CHUV) and University of Lausanne (UNIL), Neuroscience Research Center (CRN), Laboratory of Cellular and Molecular Neurotherapies (LCMN), Lausanne 1011, Switzerland; Lausanne University Hospital (CHUV) and University of Lausanne (UNIL), Department of Clinical Neurosciences (DNC), Laboratory of Cellular and Molecular Neurotherapies (LCMN), Lausanne 1011, Switzerland; Lausanne University Hospital (CHUV) and University of Lausanne (UNIL), Neuroscience Research Center (CRN), Laboratory of Cellular and Molecular Neurotherapies (LCMN), Lausanne 1011, Switzerland; Lausanne University Hospital (CHUV) and University of Lausanne (UNIL), Department of Clinical Neurosciences (DNC), Laboratory of Cellular and Molecular Neurotherapies (LCMN), Lausanne 1011, Switzerland; Lausanne University Hospital (CHUV) and University of Lausanne (UNIL), Neuroscience Research Center (CRN), Laboratory of Cellular and Molecular Neurotherapies (LCMN), Lausanne 1011, Switzerland; Lausanne University Hospital (CHUV) and University of Lausanne (UNIL), Department of Clinical Neurosciences (DNC), Laboratory of Cellular and Molecular Neurotherapies (LCMN), Lausanne 1011, Switzerland; Lausanne University Hospital (CHUV) and University of Lausanne (UNIL), Neuroscience Research Center (CRN), Laboratory of Cellular and Molecular Neurotherapies (LCMN), Lausanne 1011, Switzerland; Center for Applied Medical Research, University of Navarra, Pamplona 31008, Spain; Institute of Health Research of Navarra (IdiSNA), Cancer Center Clínica Universidad de Navarra (CCUN), Pamplona 31008, Spain; Lausanne University Hospital (CHUV) and University of Lausanne (UNIL), Department of Clinical Neurosciences (DNC), Laboratory of Cellular and Molecular Neurotherapies (LCMN), Lausanne 1011, Switzerland; Lausanne University Hospital (CHUV) and University of Lausanne (UNIL), Neuroscience Research Center (CRN), Laboratory of Cellular and Molecular Neurotherapies (LCMN), Lausanne 1011, Switzerland

**Keywords:** Huntington’s disease, gene editing, viral vectors, *HTT* inactivation, neural stem cells

## Abstract

Huntingtin-lowering strategies are central to therapeutic approaches for Huntington’s disease. Recent studies reported the induction of age- and cell type-specific phenotypes by conditional huntingtin knockout, but these experimental conditions did not precisely mimic huntingtin-lowering or gene-editing conditions in terms of the cells targeted and brain distribution, and no transcriptional profiles were provided. Here, we used the adeno-associated delivery system commonly used in CNS gene therapy programmes and the self-inactivating KamiCas9 gene-editing system to investigate the long-term consequences of wild-type mouse huntingtin inactivation in adult neurons and, thus, the feasibility and safety of huntingtin inactivation in these cells. Behavioural and neuropathological analyses and single-nuclei RNA sequencing indicated that huntingtin editing in 77% of striatal neurons and 16% of cortical projecting neurons in adult mice induced no behavioural deficits or cellular toxicity. Single-nuclei RNA sequencing in 11.5-month-old animals showed that huntingtin inactivation did not alter striatal-cell profiles or proportions. Few differentially expressed genes were identified and Augur analysis confirmed an extremely limited response to huntingtin inactivation in all cell types. Our results therefore indicate that wild-type huntingtin inactivation in adult striatal and projection neurons is well tolerated in the long term.

## Teaser

Regio *et al*. report that long-term inactivation of wild-type huntingtin in striatal and cortical projecting neurons from adult mice is well tolerated.

## Graphical Abstract

**Graphical Abstract:**
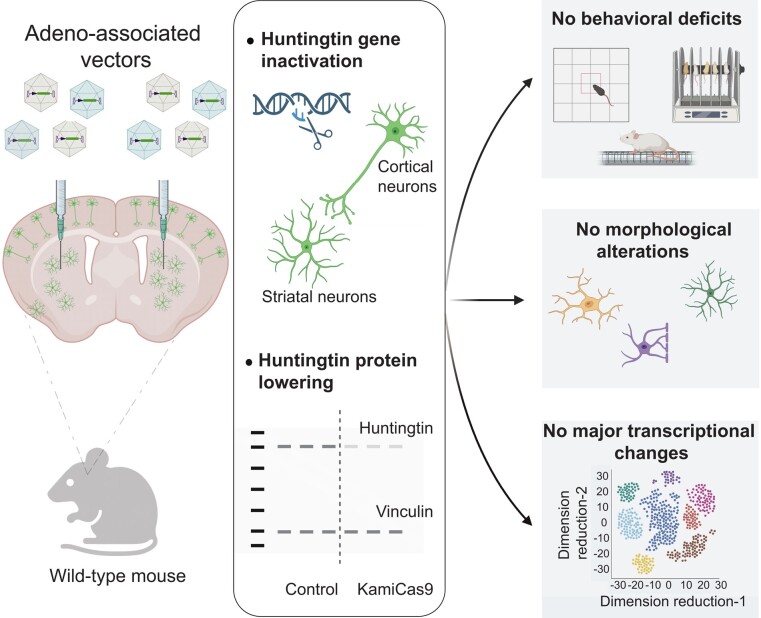

## Introduction

Huntington’s disease (OMM: 143100) is an autosomal dominant neurodegenerative disorder resulting primarily from a toxic gain of function of the HTT protein. *HTT* (OMIM: 613004)-lowering strategies have therefore been the major focus of therapeutic developments in recent years.^1^ The ultimate goal is to reduce *HTT* mRNA and/or protein levels to prevent most downstream pathogenic events. Numerous studies in mouse models of Huntington's disease have demonstrated that *HTT-*lowering approaches improve the behavioural, neuropathological and molecular features of Huntington's disease.^2^ However, the disappointing results of recent clinical trials with antisense oligonucleotides (ASOs) have served to remind us that while these approaches can offer unique opportunities for treating patients with Huntington's disease, our understanding of this disease remains partial, with many important questions still unanswered.

Various techniques are used to reduce or abolish wild-type and/or mutant *HTT* expression (allele-specific or non-allele-specific approaches). ASO, RNA interference (RNAi), Cas13d and zinc-finger repressors are used to target *HTT* transcripts themselves or *HTT* transcription, whereas CRISPR/Cas9 strategies target the *HTT* gene.^3,4^ These two types of approach have different outcomes, with *HTT*-lowering strategies decreasing *HTT* mRNA levels, whereas complete inactivation may be possible with CRISPR/Cas9 gene editing. These strategies also differ in terms of the selective targeting of the mutant *HTT* or non-allele-specific *HTT* lowering, mode of delivery, reversibility, distribution within the brain and the cells targeted.

The many open questions linked to these therapeutic strategies include the role of the wild-type HTT and the potential consequences of partially decreasing or completely abolishing wild-type *HTT* expression. Huntington's disease patients homozygous for the pathogenic allele, loss-of-function variants in the general population and experimental Huntington's disease models provide important information, but they do not precisely mimic *HTT*-lowering or gene-editing conditions. Despite these limitations, we know that homozygous Huntington's disease patients without wild-type HTT remain symptom-free for many years and that individuals with a loss-of-function (LoF) HTT variant have no clinical phenotype.^5-7^ Conversely, individuals with two *HTT* LoF alleles have a neurodevelopmental phenotype^8^ and *HTT*-knockout mice die *in utero*.^9-11^ A follow-up study in KO mice identified extra-embryonic tissues as the region primarily affected by the absence of HTT, which resulted in defective nutrient exchange in the early stages of embryonic development.^12^ Reiner *et al*.^13,14^ showed that Hdh-null cells (Hdh^−/−^) can survive in the mouse brain and that the specific distribution of Hdh^−/−^ cells in adult chimeric mice primarily reflects the differential vulnerability of Hdh^−/−^ cells in various brain regions. Finally, studies of mice with a ubiquitous or neurospecific conditional knockout of *HTT* (CAG or Nestin-CreER mice) have shown that a loss of wild-type HTT in the adult mouse brain is not associated with behavioural deficits or a neuropathological phenotype.^15^ In a second study, hemizygous mice with 50% wild-type HTT were used to generate conditional knockout (cKO) mice.^16^ However, as the level of *HTT* expression was reduced during development, it is not possible to draw conclusions about the consequences of *HTT* inactivation in adults.

All these findings suggest that the outcome of wild-type *HTT* silencing, or inactivation is age- and cell type-specific and that additional studies are required to determine more accurately the biosafety and long-term impact of lower or inactivating wild-type *HTT* expression in the adult brain. Here, we used the self-inactivating AAV-KamiCas9 gene-editing system to inactivate the wild-type *Htt* in the striatum and projecting areas in adult FVB mice. We assessed the long-term consequences of this inactivation in behavioural and neuropathological analyses and by snRNA-seq on the striatum.

## Materials and methods

### Plasmids

All plasmids used in this still are described in the Supplementary Material.

### HEK293T cell culture and transfection

HEK293T cells (mycoplasma-negative, ATCC, LGC Standards GmbH, Wessel, Germany) were cultured in DMEM-Glutamax supplemented with 10% foetal bovine serum (FBS, Gibco, Life Technologies, Zug, Switzerland) and 1% penicillin/streptomycin (Gibco, Life Technologies, Zug, Switzerland) at 37°C, under an atmosphere containing 5% CO_2_. For routine culture, cells were passaged twice weekly, with trypsin treatment for dissociation (Gibco, Life Technologies, Zug, Switzerland), and the medium was replaced every three days. Detailed conditions for each transfection are described in the Supplementary Material.

### Primary striatal cocultures and transduction

For the culture of striatal neurons, E15 C57BL/6 mouse pups (Charles River, Ecully, France) were killed by decapitation. Striatal tissue was removed and cut into small pieces, and mechanically dissociated by repeated aspiration in a fire-polished Pasteur pipette in dissecting medium [PBS without Ca^2+^ and Mg^2+^ (Life Technologies, Zug, Switzerland), 0.6% D-glucose (Sigma-Aldrich, Buchs, Switzerland), 1 mg/mL BSA (Sigma-Aldrich, Buchs, Switzerland), 10 mM HEPES (Sigma-Aldrich, Buchs, Switzerland) and 1% penicillin/streptomycin (Life Technologies, Zug, Switzerland)]. The dissociated striatal neurons were centrifuged at 300 × *g* for 5 min at 4°C, and the cell pellet was resuspended in 1 mL Neurobasal medium (Life Technologies, Zug, Switzerland) complemented with 15 mM KCl, 0.5 mM L-glutamine (Life Technologies, Zug, Switzerland), B27 (Life Technologies, Zug, Switzerland) and 1% penicillin/streptomycin. The cells obtained from seven pups were then plated at a density of 250 000 cells/well in 24-well plates containing coverslips previously coated with 50 µg/mL poly-D-lysine (Corning, Amsterdam, Netherlands) and 5 µg/mL laminin (Sigma-Aldrich, Buchs, Switzerland). Further details are provided in the Supplementary Material.

### Culture and transduction of neuronal precursor cells (NPCs)

**Figure 1:**
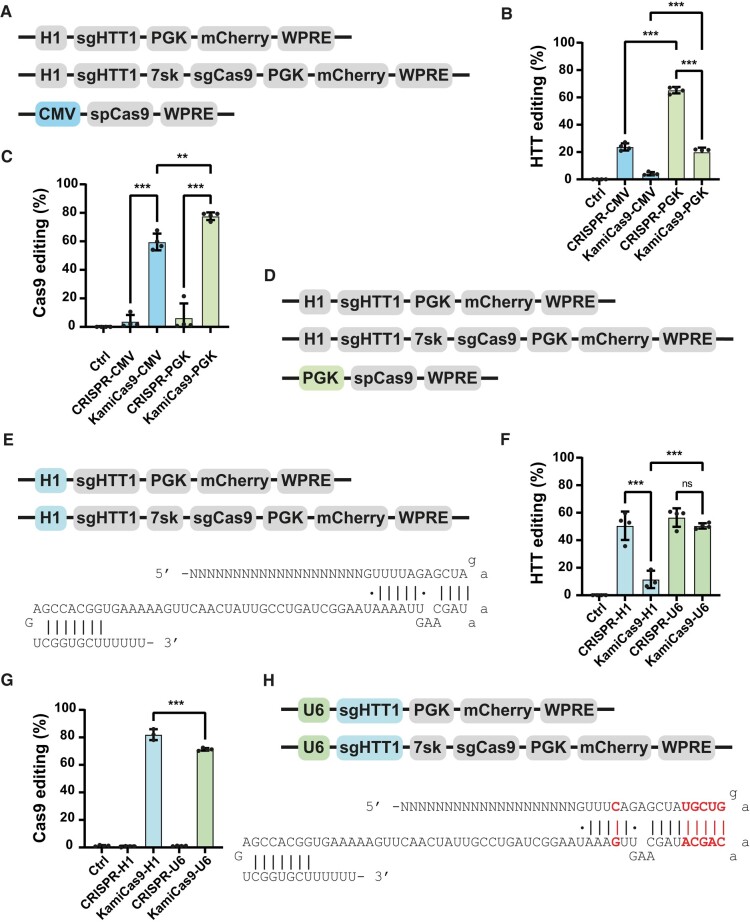
**Optimization of the LV-KamiCas9 system in human neuronal progenitor cells (NPCs).** (**A**) Schematic representation of the original constitutive and KamiCas9 vectors used for *in vitro* gene editing. The first LV expresses the sgRNA targeting the human HTT (sgHTT1) under the control of the H1 polymerase III promoter and mCherry under the control of the mouse phosphoglycerate kinase I promoter (PGK). The second vector is the KamiCas9 version with an additional cassette expressing a sgRNA targeting the translation start site of spCas9 under the control of the 7sk promoter. The third plasmid encodes the spCas9 nuclease under the control of a cytomegalovirus (CMV) promoter. (**B**) Human *HTT* editing was assessed three weeks after the transduction of NPCs. DNA extracts were used for TIDE analysis (*n* = 4 samples/group) and to compare the performances of the constitutive clustered regularly interspaced short palindromic repeats (CRISPR) and KamiCas9 systems. (**C**) The frequency of indels in the SpCas9 transgene was assessed in the same samples (*n* = 4 samples/group). (**D**) Schematic representation of the second-generation LV-KamiCas9 system. In this version, the only modification is the replacement of the CMV promoter with a PGK promoter to drive expression of the spCas9 nuclease gene. (**E**) Schematic representation of the first-generation LV-KamiCas9 system. In this version, the expression of the sgRNA targeting *HTT* is under the control of the H1 promoter and the original tracrRNA. (**F and G**) We assessed human *HTT* and spCas9 editing with the optimized system in NPCs (*n* = 4 samples/group). (**H**) Schematic representation of the final optimized LV-KamiCas9 system. In this optimized version, the expression of the sgRNA targeting *HTT* is under the control of the U6 promoter and an optimized tracrRNA. Results are presented as the mean ± SD. Statistics: one-way ANOVA and multiple comparisons with Tukey correction: ****P < 0.0001; ***P < 0.001; **P < 0.01; *P < 0.05.

Immature neuronal stem cells were generated from hPSCs line i90cl16 and provided by Dr. Anselme Perrier (CEA, Fontenay-aux-Roses, France) (Fig. 1). The differentiation and culture protocols used have been described elsewhere.^17,18^ After amplification, neuronal precursor cells (NPCs, MJ66) were banked in vials of 4 × 10^6^ cells and stored in liquid nitrogen. In the context of each experiment, cells were defrosted and resuspended in N2B27 medium (N2B27: 1:1 DEM-F12-Glutamax: Neurobasal, 1% N2 supplement, 2% B27 supplement and 0.1% gentamicin; Gibco, Life Technologies, Zug, Switzerland). Cells were centrifuged at 300 × *g* for 5 min to remove any trace of FBS and DMSO. The cell pellets were resuspended in 1 mL N2B27 medium and counted with a haemocytometer. Cells were plated at a density of 1.5–2 × 10^6^ cells per dish/well, in 35 mm dishes or six-well plates, or at a density of 1 × 10^7^ cells per dish in 10 cm dishes, in N2B27 medium supplemented with 10 ng/mL FGF (Life Technologies, Zug, Switzerland). Before plating, the dishes were coated by incubation with 17 µg/mL poly-L-ornithine (1/6 Sigma-Aldrich, Buchs, Switzerland) in H_2_O for 24 h and then by incubation with 2 µg/mL laminin (Sigma-Aldrich, Buchs, Switzerland) in H_2_O for 24 h. The medium was entirely replaced twice weekly, and the cells were passaged once per week.

Detailed conditions for each experiment are described in the Supplementary Material.

### Immunofluorescence

#### HEK293T cells (six wells)

**Figure 2:**
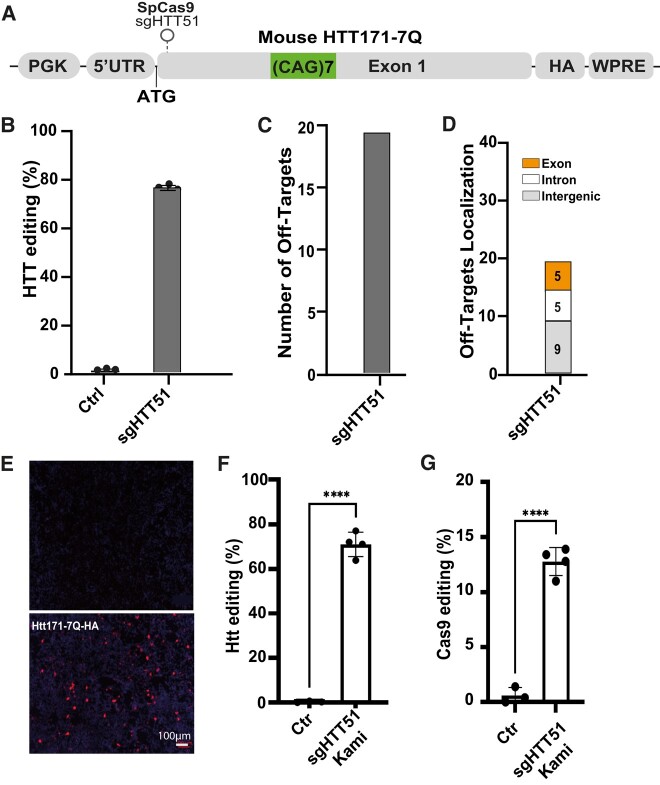
**Design and validation of sgRNA inactivating the mouse *Htt* gene.** (**A**) Schematic representation of the mouse *Htt* reporter plasmid used for validation of the various sgRNAs. The plasmid contains the 5′ untranslated region (5′UTR) part of the *Htt* gene and the codons encoding the first 171 amino acids, including the seven glutamine (codons CAG or CAA) repeats ((CAG)2CAA(CAG)4). The expression of this transgene is under the control of the PGK promoter. An HA-tag was in the 3′ region was used to facilitate the detection of this N-terminal fragment of the Htt protein by western blotting. Finally, the woodchuck post-regulatory element (WPRE) was used to increase transgene expression. The position of the sgRNA targeting the translation start site of the mouse Htt is indicated: sgHTT51. (**B**) Editing efficiency of sgHTT51 according to TIDE, 3 days after the transfection of HEK293T cells (*n* = 3 samples/group). (**C**) A bioinformatic analysis was performed to evaluate the presence of potential off-target (OT) sites for sgHTT51 binding in exons, introns and intergenic regions of the mouse genome. (**D**) In total, 19 off-target sites were detected for sgHTT51. All those with the most relevant scores were located in intronic or intergenic regions. (**E**) Immunofluorescence analysis with a mouse monoclonal HA antibody showing the loss of the mouse Htt protein following gene editing in HEK293T cells. (**F and G**) Final validation of the second-generation KamiCas9 system in mouse primary striatal cultures transduced with the corresponding lentiviral vectors. Very efficient editing of the endogenous mouse *Htt* gene (**F**) and *Streptococcus pyogenes* Cas9 (spCas9) nuclease (**G**) was observed 12 days post-transduction (*n* = 4 samples/group). Results are presented as the mean ± SD. Statistics: two-tailed *t*-test (HTT: *t*(21.91), *df*(5)5; Cas9: *t*(14), *df*(5)). ****P < 0.0001; ***P < 0.001; **P < 0.01.

The cells were washed once with PBS (Gibco, Life Technologies, Zug, Switzerland). They were then fixed by incubation in 4% paraformaldehyde (PFA, Electron Microscopy Sciences, Hatfield, USA) in 0.15 M sodium phosphate buffer (Sigma-Aldrich, Buchs, Switzerland) at 4°C for 15 min. The PFA solution was removed, and the cells were washed three times with 1× PBS (Laboratorium Dr Bichsel AG, Interlaken, Switzerland). For the visualization of fluorescence, cells were cultured on coated coverslips (50 µg/mL poly-D-lysin; Corning, Amsterdam, Netherlands) and mounted directly on Superfrost+ microscope slides, in Vectashield mounting medium with DAPI (Vector Lab Inc., Burlingame, USA) (Fig. 2E and F). For immunofluorescence staining, cells were washed three times, for 1 min each, with 1× PBS and blocked by incubation for 1 h in 1× PBS supplemented with 5% normal goat serum (NGS, Interchim, Montluçon, France) and 0.1% Triton X-100 (Fluka, Sigma-Aldrich, Buchs, Switzerland) or supplemented with 1% bovine serum albumin (BSA, Sigma-Aldrich, Buchs, Switzerland). The primary antibody, mouse monoclonal anti-HA antibody (1/1000, Lucerna-Chem AG, Luzern, Switzerland), was diluted in the blocking solution and incubated overnight with the cells at 4°C (Fig. 2E). The following day, the cells were washed three times, for 1 min each, with 1× PBS and incubated for 1 h at room temperature with secondary antibodies diluted in the blocking solution. The secondary antibody used was goat anti-mouse IgG AlexaFluor-594 (1/1000, Invitrogen, Life Technologies, Zug, Switzerland). The cells were then washed three times, for 1 min each, in 1× PBS. The coverslips were mounted on Superfrost+ microscope slides in Vectashield mounting medium for fluorescence supplemented with DAPI (Vector Lab Inc., Burlingame, USA) (Fig. 2E).

### Production of viral vectors

LVs were produced in HEK293T cells, with the four-plasmid system, as previously described.^19^ AAV were produced in HEK293T cells transfected with pHelper, pAAV2-retro_Rep_Cap, pAAV2/1_Rep_Cap, pAAV2/10_Rep_Cap and the transgene by the calcium phosphate precipitation method, as previously described.^20^

### Animals

Adult female mice (8–15 weeks old) were used for the *in vivo* experiments. The wild-type C57BL/6 or FVB mice were obtained from Janvier (Le Genest-Saint-Isle, France). Mice were housed in a specific pathogen-free facility, in individually ventilated cages GM500 (Tecniplast) or rat R.BTM.U x/R.ICV.6 cages (Innovive, Paris, France) and single-sided rat Innoracks (cat# RS.5.8.40) containing corn cob bedding. Each cage contained no more than five mice. The animals were maintained in a controlled-temperature room (22 ± 1°C) under a 14 h light/10 h dark cycle. The environment was enriched with two pieces of wipes, one cardboard tunnel, one cardboard or polysulfone house with two entrances/exits. Food (SAFE® 150, Safe, Rosenberg, Germany) and water were available *ad libitum*. All experimental procedures were performed in strict accordance with Swiss regulations concerning the care and use of laboratory animals (veterinary authorizations 3447 and 3682).

### Surgery

Mice were anaesthetized by the intraperitoneal injection of a mixture of 50 mg/kg ketamine (Sintetica, Mendrisio, Switzerland) and 10 mg/kg xylazine (Rompun, Bayer Health Care, Uznach, Switzerland). Briefly, the head of the mouse was shaved, and the mouse was placed in a stereotaxic frame (model 963 Ultra Precise Small Animal Stereotaxic Instrument, Kopf, Tujunga, USA). The body temperature of the mouse was monitored and kept constant with a heating pad (>36°C) throughout the surgical procedure. The scalp was disinfected with liquid povidone-iodine before incision and exposure of the skull. A 25G needle fixed to a syringe was used to pierce the skull at the appropriate coordinates. Suspensions of virus were injected with a 34-gauge blunt-tip needle (Unimed, Lausanne, Switzerland) linked to a Hamilton syringe (Hamilton Medical AG, Bonaduz, Switzerland), via a polyethylene catheter (Unimed, Lausanne, Switzerland). Detailed conditions for each experiment are described in the Supplementary Material.

### Behavioural analysis

After the stereotaxic surgery (Ctr versus HTT-KamiCas9), the 12-week-old mice were randomly assigned to the procedures and housed with three animals per cage, according to treatment group. Neurological assessments and behavioural testing were performed for 34 mice (*n* = 17/group) at the age of nine months. The individuals responsible for animal care and the experimenters performing the behavioural tests were blind to treatment group. A detailed description of each behavioural analysis is provided in the Supplementary Material.

### DNA extraction

#### HEK293T cells

The culture medium was removed from the six-well plates, and the cells were washed once with PBS. Cells were detached by treatment with trypsin and 500 μL PBS was added to the wells. We then transferred 40 to 60 μL of the resulting cell suspension to a 1.5 mL Eppendorf tube and centrifuged for 5 min at 300 × *g* at 4°C. The PBS was discarded and 100 μL QuickExtract DNA extraction buffer was added to the pellet. The cells were incubated at 65°C for 15 min and then at 98°C for 2 min to inactivate the lysis buffer.

#### Brain punches

We extracted gDNA from the mice as follows. The entire procedure was performed in DNAse-free conditions, in a clean area free from plasmids and PCR products, to prevent sample contamination. Mice were killed by an overdose of sodium pentobarbital (B-Braun Mediacal SA, Sempach, Switzerland) and the brain was quickly removed from the head. The isolated brain was placed in a dissection matrix and sliced into 1 mm-thick sections. Slices were transferred to glass microscope slides on ice and observed under a Nikon SMZ18 epifluorescence stereomicroscope. For all experiments, the GFP-positive area was isolated with a scalpel and each of these striatal punches was placed in an Eppendorf tube containing 100 μL QuickExtract DNA extraction buffer. Tissue punch samples were homogenized with a tissue homogenizer, vortexed for 10 s and incubated at 65°C for 15 min with shaking and then for 2 min at 98°C to inactivate the lysis buffer.

We determined the concentration of gDNA with a Nanodrop spectrophotometer (Thermo Fisher Scientific, Reinach, Switzerland). Sample concentration was adjusted to 100 ng/μL and the samples were stored at 4°C for immediate analyses. The remaining gDNA stock was stored at −20°C.

### RNA extraction

#### Brain punches

RNA was extracted from the GFP-positive striatal area in 500 μL Trizol reagent (Life Technologies, Zug, Switzerland). Briefly, after tissue homogenization and lysis, RNA was precipitated from the aqueous phase and purified in accordance with the manufacturer’s instructions. For RNA extraction, we added 5 μL RNase-free glycogen (Ambion, Thermo Fisher Scientific, Reinach, Switzerland) as a carrier, to facilitate visualization of the RNA pellet. The final RNA pellet was then resuspended in nuclease-free water (Gibco, Life Technologies, Zug, Switzerland) by passive homogenization and vortexing. RNA concentration was determined with a Nanodrop spectrophotometer (Thermo Fisher Scientific, Reinach, Switzerland). The entire procedure was performed in DNase-free conditions, in a clean area of the laboratory free from plasmids and PCR products, to prevent sample contamination. We then treated 1 µg RNA with RNase-free DNase (RQ1, Promega, Dubendorf, Switzerland) in accordance with the manufacturer’s protocol, to remove any trace of genomic DNA, and diluted the final sample to a concentration 20 ng/μL. We generated cDNAs from 200 ng DNase-treated RNA with Superscript II (Thermo Fisher Scientific, Reinach, Switzerland) in accordance with the manufacturer’s instructions. The concentration of the cDNA solution obtained was then adjusted to 1 ng/μL.

### TIDE analysis

We used 100 ng gDNA for an amplification reaction with the KAPA HiFi Hotstart kit (KAPA Biosystems, Labgene, Chatel-Saint-Denis, Switzerland). The reaction mixture contained 1×KAPA HiFi buffer, 0.3 mM dNTP mix, 0.3 μM forward and reverse primers, 0.5 U KAPA HiFi HotStart DNA polymerase, 100 ng gDNA and PCR-grade water q.s.p 25 μL and the cycle parameters were as follows: 95°C for 180 s, then 30 cycles of 98°C for 20 s, incubation at the annealing temperature for 15 s, 72°C for 30–90 s (depending on the primer pair), 72°C for 180 s, and then cooling to 4°C. The primers and PCR conditions are described in the Supplementary Material.

The sequencing data were analysed with the tracking of indels by decomposition (TIDE) method.^21^ Alignments were performed with the standard Smith–Waterman local alignment method implemented in the BioStrings package in Bioconductor. TIDE software uses the peak heights for each base, as determined by the sequence analysis software provided by the manufacturer of the capillary sequencing equipment, to determine the relative abundance of aberrant nucleotides over the length of the whole sequence trace. The R functions of TIDE were provided directly by the authors and were used to analyse the sequencing results. The indel size range was set to 10 and the size of the decomposition window was adapted for reads of low quality or containing repetitive sequences. The significance cut-off was set at *P* = 0.05 (https://tide.nki.nl/).

### Capillary-based western blotting

Protein was extracted from striatal punches (*n* = 2 Ctr mice, *n* = 4 punches; and *n* = 3 HTT-KamiCas9 mice, *n* = 6 punches) in RIPA buffer (Sigma-Aldrich, Buchs, Switzerland) supplemented with a 1/200 dilution of protease inhibitor cocktail (Sigma-Aldrich, Buchs, Switzerland) and 5 µM Z-VAD-FMK (Chemie Brunschwig, Basel, Switzerland). Protein concentration was assessed with a BCA kit (Thermo Fisher Scientific, Reinach, Switzerland) according to the recommended procedure. After dilution of the total protein extract in 0.1× SB (ProteinSimple, Bio-Techne), we subjected 0.6 μg of total protein to size separation with the Jess capillary-based immunoassay system. Samples were processed according to the manufacturer’s instructions, with the 66–440 kDa separation module (SM-W008). The antibodies used are described in the Supplementary Material.

### Histological processing

The mice were killed by an overdose of sodium pentobarbital (B-Braun Mediacal SA, Sempach, Switzerland) and transcardially perfused at a rate of 20 mL/min with 1× PBS for 2 min and then with 4% PFA for 4 min. Brains were removed and post-fixed by incubation in 4% PFA for 12 h at 4°C. They were then cryoprotected by incubation in 20% sucrose (Sigma-Aldrich, Buchs, Switzerland) in 1× PBS for 6 h and then in 30% sucrose in 1× PBS for 24 h. Brains were stored at −80°C until use. We cut 25 μm-thick coronal brain sections on a cryostat with a freezing stage at −20°C (Leica CM1850, Biosystems Switzerland, Nunningen, Switzerland). Slices throughout the striatum were collected and stored in a 96-well plate, as free-floating slices in anti-freeze solution [25% glycerol (Sigma-Aldrich, Buchs, Switzerland), 30% ethylene glycol (Merck, Nottingham, UK), 25% 1× PBS and 20% nanopure water]. Slices for direct fluorescence analysis and visualization were mounted directly on Superfrost+ microscope slides, in Vectashield mounting medium supplemented with DAPI (Vector Lab Inc., Burlingame, USA). The experimental conditions for the immunofluorescence analysis are described in the Supplementary Material.

### Nucleus isolation from frozen brain tissue, library preparation and sequencing

A Chromium Next GEM Chip M (10X genomics) was loaded with the appropriate number of cells, and sequencing libraries were prepared with the Chromium Next GEM Single Cell 3′ HT Kit v3.1, in strict accordance with the manufacturer’s recommendations. Briefly, we generated an emulsion of single cells, reverse transcription reagents and cell-barcoding oligonucleotides. Following the reverse transcription step, the emulsion was disrupted and double-stranded cDNAs were generated and amplified in a bulk reaction. The cDNA was fragmented and ligated to a P7 sequencing adaptor, and a 3′ gene expression library was generated by PCR amplification. Libraries were quantified by a fluorimetric method and their quality was assessed on a Fragment Analyzer (Agilent Technologies). Sequencing was performed on Illumina NovaSeq 6000 v1.5 flow cells for 28-10-10-90 cycles (read1 - index i7 - index i5 - read2) with a 1% PhiX spike-in. Sequencing data were demultiplexed with bcl2fastq2 Conversion Software (v. 2.20, Illumina) and primary data analysis was performed with the Cell Ranger Gene Expression pipeline (version 7.1.0, 10X Genomics). The scRNA-seq analysis is described in the Supplementary Material.

### Statistical analysis

Data are presented as the mean ± standard deviation (SD). Statistical analyses were performed with GraphPad software (GraphPad Prism version 8.00 for Windows, GraphPad Software, La Jolla, CA, USA, www.graphpad.com). Unpaired Student’s *t*-tests were used for pairwise group comparisons and nested *t*-tests for the analysis of multiple punches isolated from individual animals. For comparisons of more than two groups, we performed one-way analysis of variance (ANOVA) followed by Tukey *post hoc* correction. We checked that the data met the requirements for ANOVA of a normal distribution, with equal variances. Figures were generated with GraphPad software (GraphPad Prism version 8.00 for Windows, GraphPad Software, La Jolla, CA, USA, www.graphpad.com).

#### Behavioural analysis

Data for the two groups of mice were compared in unpaired *t*-tests, once assumptions of equal variances and a normal distribution were verified. For datasets violating these parametric assumptions (e.g. coat hanger data), groups were compared in Mann–Whitney U-tests. None of these null-hypothesis tests provided evidence of an effect at an alpha risk of 0.05. We therefore performed a series of two-one-sided *t*-tests to check for data equivalence. For each variable, we assumed equivalence for thresholds of 20% or 30% above or below the mean values of the control groups, as shown in Supplementary Table 1. Equivalence thresholds of 30% were chosen when the standard deviations were of similar magnitude to the mean values.

### Data availability

The accession number for the RNA-seq data reported here is NCBI Bioproject: PRJNA949431.

## Results

### Optimized lentiviral-KamiCas9 system

We previously developed the KamiCas9 gene-editing technology that is a self-inactivating system based dual lentiviral vectors.^22^ We demonstrated efficient *HTT* editing *in vitro* and *in vivo* with few off-target events due to *Streptococcus pyogenes* Cas9 (spCas9) inactivation. However, the on-target activity of the KamiCas9 system was slightly lower than that of the constitutive system. We hypothesized that the kinetics of *HTT* and *spCas9* editing were suboptimal, leading to premature inactivation of the spCas9 nuclease. We therefore improved the KamiCas9 system further, by replacing the cytomegalovirus (CMV) promoter driving the expression spCas9 with the phosphoglycerate kinase 1 (PGK) promoter (Fig. 1A and B). We used the previously described single guide HTT1 (sgHTT1) and sgCas9 targeting the translational start sites of the human HTT and spCas9 genes, respectively.^22^ Editing efficiency was assessed in human neural progenitor cells (NPCs) transduced with these two KamiCas9 systems and the corresponding constitutive versions (without sgCas9, hereafter referred to as CRISPR) (Fig. 1C and D). As a negative control, NPCs were transduced with a lentiviral vector expressing the sgRNAs. The TIDE method was used to measure small insertions or deletions (indels).^21^ An analysis performed three weeks post-transduction revealed 2.4- and 2.9-fold increases in *HTT* editing rates when the PGK promoter was used to drive the expression of *spCas9* in the CRISPR and KamiCas9 systems, respectively (Fig. 1C). However, *HTT* editing remained suboptimal (20.0 ± 3.24%) relative to the constitutive PGK-CRISPR system (65.3 ± 2.32%) (Fig. 1C). In addition, Cas9 editing rates also increased from 59.6 ± 5.9% to 77.6 ± 2.67% using PGK-KamiCas9 (Fig. 1D).

We further improved the KamiCas9 system by replacing the H1 promoter with the stronger U6 promoter (Fig. 1E and F). We also modified the trans-activating crRNA (tracrRNA) sequence of the sgHTT1.^23^ This optimized tracrRNA contains an extended duplex sequence before the loop and a mutation of the fourth thymine in the continuous sequence of thymine residues, preventing the premature termination of sgRNA synthesis. Importantly, sgCas9 was not modified and remained under the control of the 7sk promoter and the original tracrRNA. We then assessed the performance of this editing platform in NPCs (Fig. 1G and H). The KamiCas9-PGK-U6 induced *HTT* editing (50.4 ± 1.99%) at levels similar to those achieved with CRISPR-PGK-H1 (50.6 ± 10.34%) and CRISPR-PGK-U6 (56.5 ± 6.71%) (Fig. 1G). Furthermore, Cas9 inactivation with the KamiCas9-PGK-U6 system reached 71.3 ± 0.94% (Fig. 1H). In summary, by combining three modifications, we generated an optimal second-generation lentiviral-KamiCas9 for *HTT* inactivation in NPCs.

### Evaluation of the sgHTT targeting the wild-type mouse *HTT*

We evaluated the impact of mouse wild-type HTT (*Htt*) inactivation *in vivo* with sgHTT51, the mouse equivalent of the human sgHTT1^22^ (one mismatch between the human and mouse sequences) (Fig. 2A). The CRISPRseek Bioconductor package was used to identify potential off-target sites^24^ (Fig. 2C and D). We identified 19 off-target sites for sgHTT51, but all were associated with a very low score (score < 2 with scores from 0–100). We previously showed that editing was undetectable for 20/21 off-target sites with sgHTT1 in iPSCs and that the KamiCas9 system decreased cleavage at OT site 1 (OT1) by 79% (0.4% of the reads).^22^ Consequently, we did not perform an off-target analysis of sgHTT51 in mouse cells. We measured editing efficiency for sgHTT51 in HEK293T cells transfected with an exogenous mouse HTT reporter plasmid containing the 5′UTR and exonic region encoding the first 171 amino acids of Htt with seven CAG repeats and the haemagglutinin tag (HA) (Fig. 2A). TIDE analysis indicated an editing rate of 76.7 ± 0.6% for the mouse *Htt* reporter gene with sgHTT51 (Fig. 2B). A corresponding loss of Htt protein was demonstrated by immunofluorescence analysis with an antibody against HA (Fig. 2E). Finally, we demonstrated a high editing efficiency for this optimized Lentiviral-KamiCas9 system in primary mouse striatal cultures (Fig. 2F and G).

### *In vivo Htt* inactivation with AAV-KamiCas9 vectors

**Figure 3:**
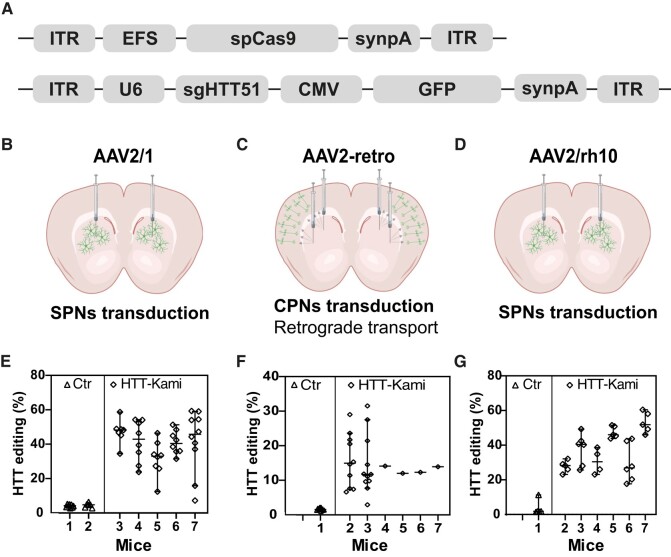
***Htt* editing in wild-type mice.** (**A**) Schematic representation of the AAV vectors used for *in vitro* gene editing. The first plasmid encodes the *Streptococcus pyogenes* Cas9 (spCas9) nuclease under the control of the short elongation factor (EFS) promoter in an AAV vector containing inverted terminal repeats (ITR) and a synthetic polyadenylation site (synpA). The second vector encodes sgHTT51 under the control of the U6 promoter and green fluorescent protein (GFP) under the control of the CMV promoter. SynpA, synthetic polyadenylation signal. (**B**) AAV2 serotype 1 (AAV2/1) (*n* = 2 Ctr, *n* = 5 treated mice), (**C**) AAV2 variant with retrograde properties (AAV2.retro) (*n* = 2 Ctr, *n* = 6 treated mice) and (**D**) AAV2 serotype 10 (AAV2/rh10) (*n* = 1 Ctr, *n* = 6 treated mice) were used to transduce spiny projection neurons (SPN) and/or cortical projection neurons (CPN), making use of the retrograde transport properties of AAV2.retro (schemes created with Biorender.com). (**E**) *Htt* editing in the striatum of mice treated with AAV2/1 (*n* = 6–8 punches/animal). Statistics: nested two-tailed *t*-test: *P* = 0.0004, *t*(8.477), *df*(5) and median with 95% CI are indicated. (**F**) *Htt* editing in the cortex of animals treated with AAV2.retro (*n =* 10 punches/mouse for three animals; and pooled punches/mouse for four animals). Statistics: nested two-tailed *t*-test: *P* < 0.0001, *t*(5.553), *df*(33)) and median with 95% CI are indicated. (**G**) *HTT* editing in the cortex of animals treated with AAV2/rh10 (*n* = 5 punches/animal) Statistics: nested two-tailed *t*-test: *P* = 0.0294, *t*(3.02), *df*(5)) and median with 95% CI are indicated.

Based on the work of Nishiyama *et al*.,^25^ we used the AAV2/1 for *in vivo* experiments. A broad distribution in the striatum (Supplementary Fig. 1A) and the selective transduction of striatal neurons were observed (Supplementary Fig. 1B). We produced the corresponding AAV2/1 vectors expressing the dual CRISPR system and assessed endogenous *Htt* editing in the striatum of C57BL6 mice (*n* = 5 treated and *n* = 2 Ctr). Animals received bilateral injections of the dual AAV2/1-EFS-spCas9 and AAV2/1-U6-sgHTT51-CMV-GFP vectors (Fig. 3A and B). They were killed four weeks after surgery; the transduced cells were identified on the basis of GFP expression and editing efficiency at the endogenous *Htt* locus was assessed by PCR and TIDE analysis. To demonstrate the potential of the approach for editing endogenous loci in large area of the striatum, several punches per mice were analysed (Fig. 3E). Strong and consistent editing of the wild-type *Htt* gene was observed in all animals and in all punches, while background values were observed in control mice (Fig. 3E). As previously observed for sgHTT1^22^ (one mismatch with sgHTT51), most edited *Htt* alleles resulted in the insertion of one adenine residue (+1A) (Supplementary Fig. 2A).

### Targeting brain circuits to inactivate *Htt*

Together the cortex and striatum are the regions of the brain most affected in Huntington's disease.^26^ However, all studies on *HTT* editing to date have focused on the striatum. We evaluated the transduction profile of AAV2.retro, to check that the transgenes were broadly distributed over the circuits affected by Huntington's disease^27^ (Fig. 3C). After intrastriatal injection, GFP-positive cells were detected mostly in cortical regions.^20^ The analysis of multiple cortical punches in two animals revealed some variability in *Htt* editing (3–29% range; Fig. 3F), which was expected based on the cortico-striatal connectome. In the last four animals, we dissected GFP-positive cortical regions in a single sample and *Htt* editing reached 13,7 ± 1,08% (Fig. 3F). Interestingly, the number of GFP-positive cells in the striatum was lower than that achieved with AAV2/1.^20^ We tried to resolve this problem by performing co-infection with AAV2/1 and AAV2.retro. However, this decreased transduction efficiency in both the cortex and striatum (Supplementary Fig. 1C). As an alternative, we used AAV2/rh.10 (Fig. 3D), which belongs to another clade of AAV.^28^ The editing efficiency in the striatum with AAV2/rh10 (Fig. 3G) was similar to that achieved with AAV2/1 (Fig. 3E) and massive transduction was observed at site of injection, in the striatum and projecting areas.^20^

### Behavioural analysis after *Htt* inactivation

**Figure 4:**
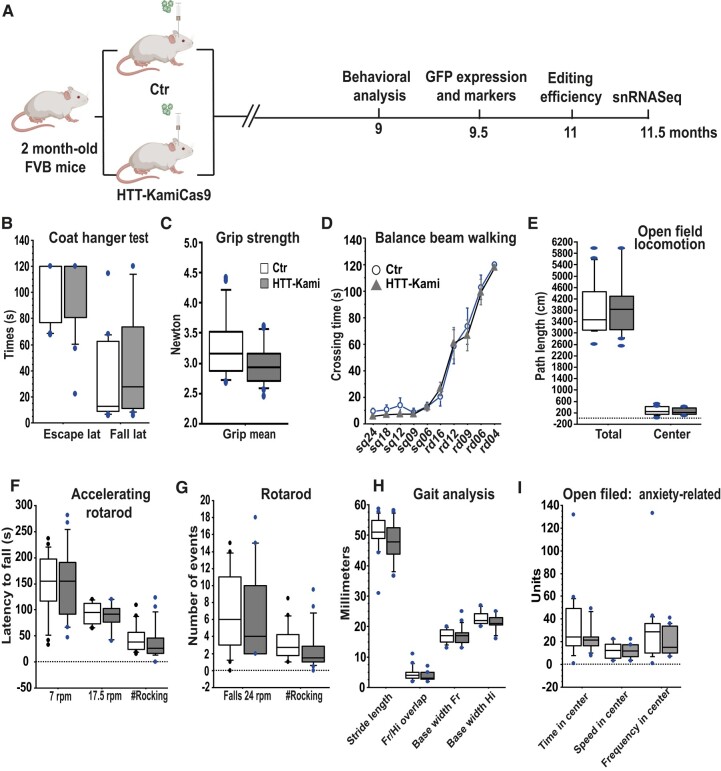
**Adult wild-type mice treated with Htt-KamiCas9 are not statistically different from their controls in behavioural analyses.** (**A**) Diagram depicting the experimental setting for the inactivation of *Htt* in wild-type mice (FVB strain) and the behavioural, neuropathological, biochemical and molecular analyses performed (*n* = 17 mice/group; created with Biorender.com). (**B**) Coat hanger test; (**C**) grip strength; (**D**) balance beam walking performed on square beams (sq) and round beams (rd) of various widths; (**E**) Open-field locomotion; (**F**) accelerating rotarod (**G**) or rotarod performance; (**H**) gait analysis (frontlimb (Fr) and hindlimb (Hi)) and (**I**) anxiety-related variables measured in open-field conditions at nine months of age. Statistics: unpaired *t*-tests, after verification of the assumptions of equal variance and normal distribution of the data. For datasets violating parametric assumptions (e.g. coat hanger data), group comparisons were performed with the Mann–Whitney U-test.

We used the AAV-kamiCas9 system to address the issue of the long-term consequences of wild-type *Htt* inactivation in adult mice.^20^ We used FVB mice, which are more susceptible to neurodegeneration than C57BL/6 mice.^29^ Two-month-old wild-type FVB mice received bilateral co-injections of AAV2/rh.10- and AAV2.retro-KamiCas9 targeting *Htt* (sgHTT51 and sgCas9_2) and a GFP-expressing vector for the identification of transduced cells. The control group received injections of all vectors other than that carrying spCas9 (Fig. 4A). Four months after surgery, a capillary-based western blot showed that Htt protein levels were 55.3 ± 9.1% lower in the striatum (*n* = 2 Ctr mice; four punches and *n* = 3 Htt-KamiCas9 mice; six punches) (Supplementary Fig. 2B and C). Behavioural analysis was performed seven months after surgery, to assess the impact of *Htt* inactivation (Ctr *n* = 17, Htt-KamiCas9 *n* = 17) (Fig. 4H). We evaluated the effect on motor function with open-field, accelerating rotarod, grip strength, beam walking and gait analyses. We also used the open-field test to assess anxiety-related behaviours through analyses of the frequency of entry, time spent and speed of movement in the centre (Fig. 4I). The Htt-KamiCas9 group did not differ significantly from the controls for any of these parameters (Fig. 4B–H). We used an equivalence test to obtain additional support for the absence of a true effect. We found that most behavioural parameters (9/16) were similar between the Ctr and Htt-KamiCas9 animals, and that none of the differences observed were significant (Supplementary Table 1), providing additional evidence for an absence of impact of wild-type *Htt* inactivation in spiny projection neurons (SPN) and cortical projection neurons (CPN) on behaviour.

### Molecular and immunochemical analysis

**Figure 5:**
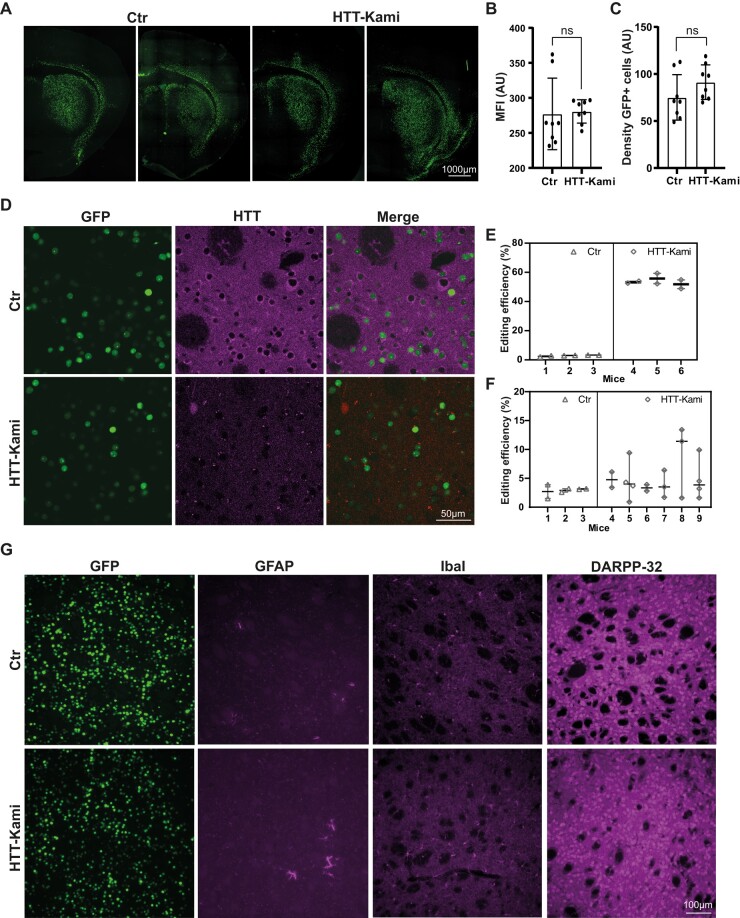
**Normal brain features following HTT inactivation in adult FVB mice.** (**A**) Two representative striatal sections from 9.5-month-old Ctr and Htt-KamiCas9 mice showing nuclear expression of the GFP encoded by the AAV vectors. (**B and C**) Quantitative analysis confirmed that the mean fluorescence intensity (MFI in arbitrary unit: AU) (**B**) and the density of GFP-positive cells (**C**) were similar in the two groups (*n* = 4 animals/group). Statistics: two-tailed *t*-test (MFI: *P* = 0.8539, *t*(0.1876), *df*(14), density: *P* = 0.1528, *t*(1.512), *df*(14)). (**D**) Confocal images showing the loss of Htt in GFP-positive striatal cells. Cortical (*n* = 3 animals/Ctr, *n* = 6/treated animals; 2–4 punches/animal) (**E**) and striatal *Htt* editing (*n* = 3 mice/group, 2 punches/animal) (**F**) in 11-month-old mice. Statistics: nested two-tailed *t*-test (cortex: *P* = 0.1658, *t*(1.433), *df*(22), striatum: *P* < 0.0001, *t*(35,5), *df*(10)). (**G**) Immunostaining for glial fibrillary acidic protein (GFAP), ionized calcium binding adaptor molecule 1 (IbaI) and dopamine- and cyclic-AMP-regulated phosphoprotein of molecular weight 32 000 (DARPP-32) in Ctr and Htt-kamiCas9 mice.

Editing rates for *Htt*, measured by TIDE (in 11-month-old animals), reached 53.6 ± 3.4% in striatal punches and 5.1 ± 3.6% in cortical punches (Fig. 5E and F). The spCas9 nuclease is expressed only in neurons. Given the known proportions of neurons in these brain structures,^20^ we can estimate that ∼77% of striatal and 16% of cortical neurons were edited. These editing data are consistent with previous findings obtained four months post-surgery.^20^ We previously showed that editing efficiency and HTT protein loss are strongly correlated in the striatum.^20^ We assessed potential deleterious effects further in six animals per group (killed at the age of 9.5 months). We determined the number of GFP-positive cells in the striatum of these control (Ctr) and Htt-KamiCas9 mice (Fig. 5A). We found no difference in mean fluorescence intensity (Fig. 5B) or density of GFP-positive cells between these two groups of mice (Fig. 5C). Confocal analysis confirmed the loss of Htt protein in GFP-positive cells (Fig. 5D). Furthermore, histological assessments based on GFP, GFAP, IbaI and DARPP-32 revealed an absence of morphological alterations in Htt-KamiCas9-treated mice (Fig. 5G). These data demonstrate a massive inactivation of wild-type *Htt* in the basal ganglia circuits in 11-month-old animals with no evidence of massive cell death or changes to transduced cells.

### Single-nuclei RNA sequencing

**Figure 6:**
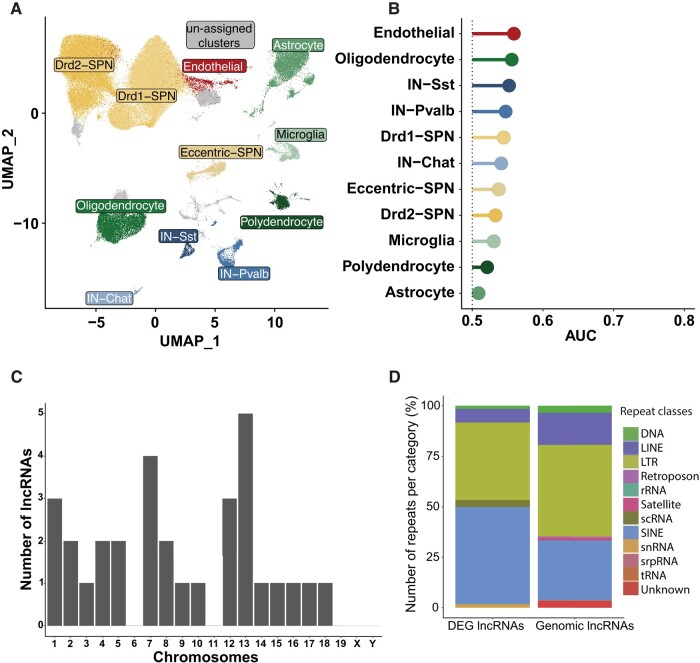
**Single-nuclei RNA-seq.** (**A**) Uniform manifold approximation and projection (UMAP) of 108 550 cells by identified cell type, from Malaiya and coworkers (*n* = 4 animals/group). D1 dopamine receptor (Drd1) or D2 dopamine receptor (Drd2) spiny projections neurons (SPN), somatostatin (SST), parvalbumin (Pvalb), and choline acetyltransferase (Chat) and interneurons (IN) were identified. (**B**) Augur analysis. The area under the receiver operating characteristics curve (AUC) is only slightly larger than that expected by chance (0.5) for all cell types, indicating an absence of major differences between the control and *HTT* KO for any cell type. (**C**) Chromosomal localization of long non-coding RNAs (lncRNAs) differentially expressed in SPNs. (**D**) Distribution of repeats and transposable elements in lncRNAs differentially expressed (DE) in SPNs relative to total genome lncRNAs.

**Figure 7:**
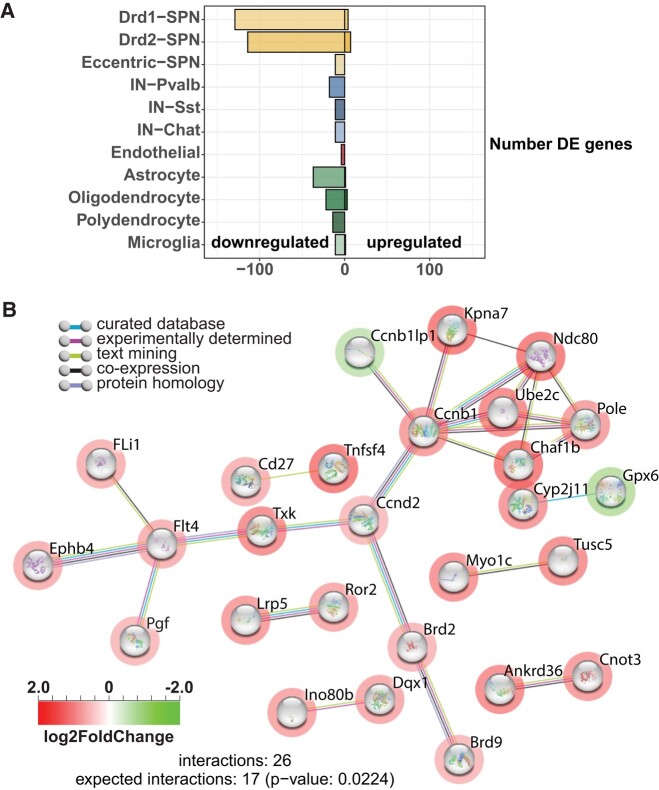
**Analysis of differentially expressed genes.** (**A**) Number of genes upregulated [right; log_2_FC ≥ 1; false-discovery rate (FDR) ≤ 0.05] and downregulated (left; log_2_FC ≤ 1; FDR ≤ 0.05) in each cell type. (**B**) Interaction network for proteins (STRING) represented by genes differentially expressed after *HTT* inactivation in dSPN. Red circles indicate downregulated genes and green circles indicate upregulated genes. rRNA, ribosomal RNA; srpRNA, signal recognition particle RNA; tRNA, transfer RNA.

HTT is an important transcriptional regulator^30^ and many studies have reported an early dysregulation of transcription in Huntington's disease patients and mouse models of Huntington's disease.^31-33^ However, no data are available for wild-type *HTT* inactivation in the adult brain. We therefore performed single-nuclei RNA sequencing (snRNA-seq) on 11.5-month-old animals. We dissected out the striatum from mice from the Ctr and Htt-KamiCas9 groups (*n* = 4/group). The samples were frozen and nuclei were isolated and sequenced (Fig. 6). Filtering and quality control procedures were applied, resulting in the retention of 108 550 high-quality nuclei for analysis. We identified 34 clusters (Supplementary Fig. 3). Cell-type assignment (iSPN-Drd2 neurons, dSPN-Drd1 neurons, Pvalb interneurons, Chat interneurons, Sst interneurons, oligodendrocytes, astrocytes, endothelial cells, microglia and polydendrocytes) was performed as previously described (Fig. 6A, Supplementary Figs 3and 4).^34,35^ We then used the Augur pipeline to identify the cell types in the striatum most responsive to neuronal *Htt* inactivation (Fig. 6B).^36^ This analysis identified the cells displaying the most marked alterations between experimental conditions in a manner unbiased by the numbers of cells in each group. The area under the receiver operating characteristics curve (AUC) was only slightly greater than expected for a random change (0.5), indicating a very limited impact of *Htt* inactivation (Fig. 6B). This limited impact was confirmed by the differential expression analysis with DESeq2 (log_2_ > 1; adjusted *P*-value 0.05) (Fig. 7A, Supplementary Fig. 5).^37^ We identified only 450 cell type-specific changes in gene expression, involving 254 genes (150 genes and 104 lncRNAs) (Supplementary Table 2). Most of these DEGs were found in the two most abundant cell types, dSPN and iSPN (166), and the vast majority (160) were downregulated, with 38% of the downregulated genes common to dSPN and iSPN. We performed pairwise comparisons, which indicated that most genes displayed changes in the same direction, consistent with the transcriptional modifications being due to *Htt* inactivation rather than random changes (data not shown). We then investigated the 31 lncRNAs downregulated in dSPN and iSPN. We analysed the presence of repeats and transposable elements, which are increasingly recognized as conserved, essential functional elements that can affect the processing, stability, localization and function of lncRNAs.^38^ We identified 60 repeats, and we found that 25 of the lncRNAs had at least one repeat. Nine of these lncRNAs were localized on chromosomes 13 and 7 (Fig. 6C). The downregulated lncRNAs included a higher frequency of short interspersed nuclear elements (SINEs) than total genomic lncRNAs, but a lower frequency of long terminal repeats (LTRs) or long interspersed nuclear elements (LINEs) (Fig. 6D). Finally, the 31 downregulated lncRNAs displayed no cell specificity (Supplementary Fig. 6). However, at this stage, more detailed characterization of the lncRNAs in the brain is required to shed light on the contribution of these lncRNAs to HTT biology.

Finally, we performed an additional analysis of the DEG in SPN. Due to the limited number of DEGs, pathway analysis was inconclusive. String analysis with default parameters indicated that dSPN were the only cell type with more interactions than expected (Fig. 7B). We then performed a manual analysis of the genes downregulated in iSPN and dSPN with no logFC cut-off and an adjusted *P*-value of <1 × 10^6^ considered significant. An evaluation of these 87 coding genes (Supplementary Table 3) revealed that 44 were potentially involved in transcriptional regulation (Aim2, Ascc2, Brd9, Esrra, Gtf3c5, MALAT1, Maml3, Med24, Phc1, Polr1a, Supt5, Wdr43, Zcchc9), RNA processing (Clk1, Cnot3, Dalrd3, Nrde2, Nsun5, Pabpc1, Pabpc4, Prpf4b, Surf6, Zc3h7b), nuclear-chromatin functions (Baz1a, Brd2, Dnase1l3, Emsy, Glis3, Ints3, Parp1, Setd1a, Ubqln4, Uhrf1bp1) or vesicular trafficking-cytoskeleton functions (Ccz1, Dync1i2, Eps8l1, Lzts1, Mast3, Mlst8, Myo1c, Myo3b, Myo18a, Nisch, Snx32). These cellular pathways are consistent with the known role of HTT as a molecular scaffold involved in multiple complexes regulating cell dynamics and gene expression.^39^

In summary, we demonstrated that AAV-KamiCas9 effectively inactivated *Htt* in the basal ganglia circuit, resulting in a modest change in the transcriptional profile of striatal cells with no overt behavioural or neuropathological consequences.

## Discussion

Studies of conditional knockout models have demonstrated that the impact of *HTT* inactivation is age- and cell type-specific.^15,16^ However, no snRNA-seq data are available for WT mice or mice in which wild-type *Htt* expression has been lowered or inactivated in the adult brain. The transcriptomics studies performed to date have been performed in mice expressing mutant *HTT*, to decipher the molecular mechanisms underlying the deleterious effects of polyQ expansion. Data for the effects of lowering or abolishing wild-type *Htt* expression are much less widespread and the normal functions of wild-type Htt remain incompletely defined. With the development of HTT-lowering programmes for the treatment of Huntington's disease patients, further studies are required to evaluate the long-term consequences of wild-type *Htt* inactivation.

Here, we inactivated wild-type *Htt* in the basal ganglia of adult mice and analysed the long-term consequences of this inactivation. We closely reproduced the experimental framework currently being developed for HTT-lowering strategies based on AAV gene therapy. Bulk editing analysis on brain punches revealed that AAV-KamiCas9 treatment led to 54% *Htt* inactivation in striatal punches, and 5% *Htt* editing in cortical samples. If we consider spCas9 to be expressed exclusively in neurons, and assume that neurons account for 65–70% of all striatal cells, then *Htt* was inactivated in 77–83% of striatal neurons.^20^ These figures demonstrate that our AAV-KamiCas9 strategy induced mostly biallelic editing of *Htt* alleles, consistent with previous reports of 99.4% biallelic editing in transduced neurons.^40^ Thus, *Htt* inactivation was induced in a large proportion of SPNs and in CPNs projecting onto these SPNs, whereas a small fraction of unedited SPNs and all other cell types (GFP-negative) continued to express *Htt*, resulting in tissue mosaicism. Capillary western blotting and Htt immunofluorescence analysis on brain sections confirmed a 53.6 ± 3.4% loss of Htt in the striatum. The residual Htt signal in the striatum is consistent with our editing efficiency, AAV transduction pattern, the presence of HTT-positive axons projecting from other brain regions^41^ and the editing profile of sgHTT51, which mostly introduced a premature stop codon in *Htt*.

We investigated the consequences of *Htt* inactivation by assessing motor functions in rotarod, grip strength, beam walking, and gait analyses and anxiety-related behaviours in open-field tests. Htt-KamiCas9-treated mice and Ctr mice did not differ significantly, and equivalence tests providing additional evidence that the means of the two groups of mice were equivalent for most parameters. We also checked that *Htt* inactivation did not alter the expression profiles of transduced cells or the expression of microglial (IbaI), astrocytic (GFAP) and neuronal markers (DARPP-32 and NeuN). Our results are consistent with those of previous studies demonstrating that a partial decrease in HTT mRNA/protein levels is well tolerated in mice and macaques^42-45^ and that the ablation of *Htt* in nestin or CAMKII neurons (CAGG-CreER, CaMKII-CreER or Nestin-CreER-mediated) in adult mice does not result in overt pathological features in the striatum.^15,16^ By contrast, conditional KO in mice with only 50% the normal level of Htt before inactivation was more deleterious. Wang *et al*. reported gait abnormalities and lower levels of locomotor activity, together with thalamic and cerebellar alterations, in such mice. However, they observed no alterations or cell death in the striatum or cortex.^16^ These results indicate that the inactivation of wild-type *Htt* is well tolerated in SPN and CPN, but other brain cells or structures, such as the thalamus, may be more vulnerable. While these data suggest that inactivation of wild-type and mutant HTT in SPN and CPN could be considered in patients with Huntington's disease, we cannot exclude the possibility that species-specific differences in life expectancy, brain structures/functions and the presence of mutant HTT in patients with Huntington's disease may alter the outcome of HTT inactivation.

We further investigated the cell type-specific signature induced by *Htt* inactivation by performing snRNA-seq on striatal samples. We used four biological replicates per group (Ctr and Htt-kamiCas9) and analysed over 108 550 nuclei after quality controls and filtering. The major cell populations of the striatum were identified in well-defined clusters. The proportion of nuclei in each cell type-specific cluster was consistent with that reported by Malaiya *et al*.^34^ Furthermore, the profile and proportions of striatal cells were not altered by *Htt* inactivation. We then used Augur, a method that focuses on the most affected cell types rather than on individual genes. The classic DEG method prioritizes abundant cell types over small clusters, even if these rarer cell types are highly affected. This is a particularly relevant factor in the striatum, where dSPN, iSPN and eccentric SPN (eSPN) account for 63% of all the nuclei analysed. Augur and DEG analyses revealed that the long-term consequences of *Htt* inactivation in the striatum were extremely limited, in all cell types. This may reflect the modest level of *Htt* inactivation in the cortex (16% of CPNs). However, this proportion corresponds to up to 45% of the neurons that could be targeted by retrograde transport in some samples.^20^ We might therefore have expected circuitry disruption and transcriptional alterations in a subpopulation of SPNs, but no such changes were observed. We found that 51% of the 87 SPN genes most altered by *Htt* inactivation were linked to cellular pathways involved in HTT biology, such as transcriptional regulation, RNA processing, nuclear-chromatin and vesicular trafficking-cytoskeleton functions. Ten of these genes also displayed alterations in expression in SPNs from 14- to 15-month-old HttQ175/+ mice, sometimes in opposite directions (Maml3, Polr1a, Rpgr, Parp1; Supplementary Table 3).^34^ Further studies are now required to determine (i) whether *Htt* inactivation results in transcriptional dysregulation via direct interaction with transcription factors and/or chromatin-modifying proteins; (ii) whether *Htt* is an upstream modulator of these pathways; (iii) the contribution of lncRNAs; and (iv) whether compensatory mechanisms or translational regulation of a subset of mRNA is involved in this limited transcriptional response.^46,47^ Independently of fundamental questions about wild-type Htt biology, our findings indicate that the long-term cortico-striatal inactivation of *Htt* is safe, with no impact on any of the behavioural and neuropathological parameters analysed, and that this inactivation induces only very limited striatal transcriptional dysregulation further supporting the use of therapeutic strategies based on the lowering of HTT levels.

## Supplementary Material

fcad344_Supplementary_Data
