# Supplementary material for: Revisiting the outcome of adult wild-type Htt inactivation in the context of HTT-lowering strategies for Huntington’s disease
Source: Brain Commun. 2023 Dec 7;5(6):fcad344. doi: 10.1093/braincomms/fcad344 (PMC10729863; doi:10.1093/braincomms/fcad344)
Supplement: fcad344_Supplementary_Data [file fcad344_supplementary_data.zip › Supplementary_material.docx]

**Supplementary data**

**Revisiting the outcome of adult wild-type HTT inactivation in the context of HTT-lowering strategies for Huntington’s disease**

Sara Regio ^1,2^**^*^**, Gabriel Vachey ^1,2^**^*^**, Enrique Goñi ^3,4^, Fabio Duarte ^1,2^, Margareta Rybarikova  ^1,2^, Mélanie Sipion ^1,2^, Maria Rey ^1,2^, Maite Huarte ^3,4^ and Nicole Déglon^1,2^

^*^Sara Regio and Gabriel Vachey contributed equally to this work.

1 Lausanne University Hospital (CHUV) and University of Lausanne (UNIL), Department of Clinical Neurosciences (DNC), Laboratory of Cellular and Molecular Neurotherapies (LCMN), Lausanne, Switzerland.

2 Lausanne University Hospital (CHUV) and University of Lausanne (UNIL), Neuroscience Research Center (CRN), Laboratory of Cellular and Molecular Neurotherapies (LCMN), Lausanne, Switzerland

3 Center for Applied Medical Research, University of Navarra, Pamplona, Spain

4 Institute of Health Research of Navarra (IdiSNA), Pamplona, Spain

**Supplementary Figures and Tables**

**
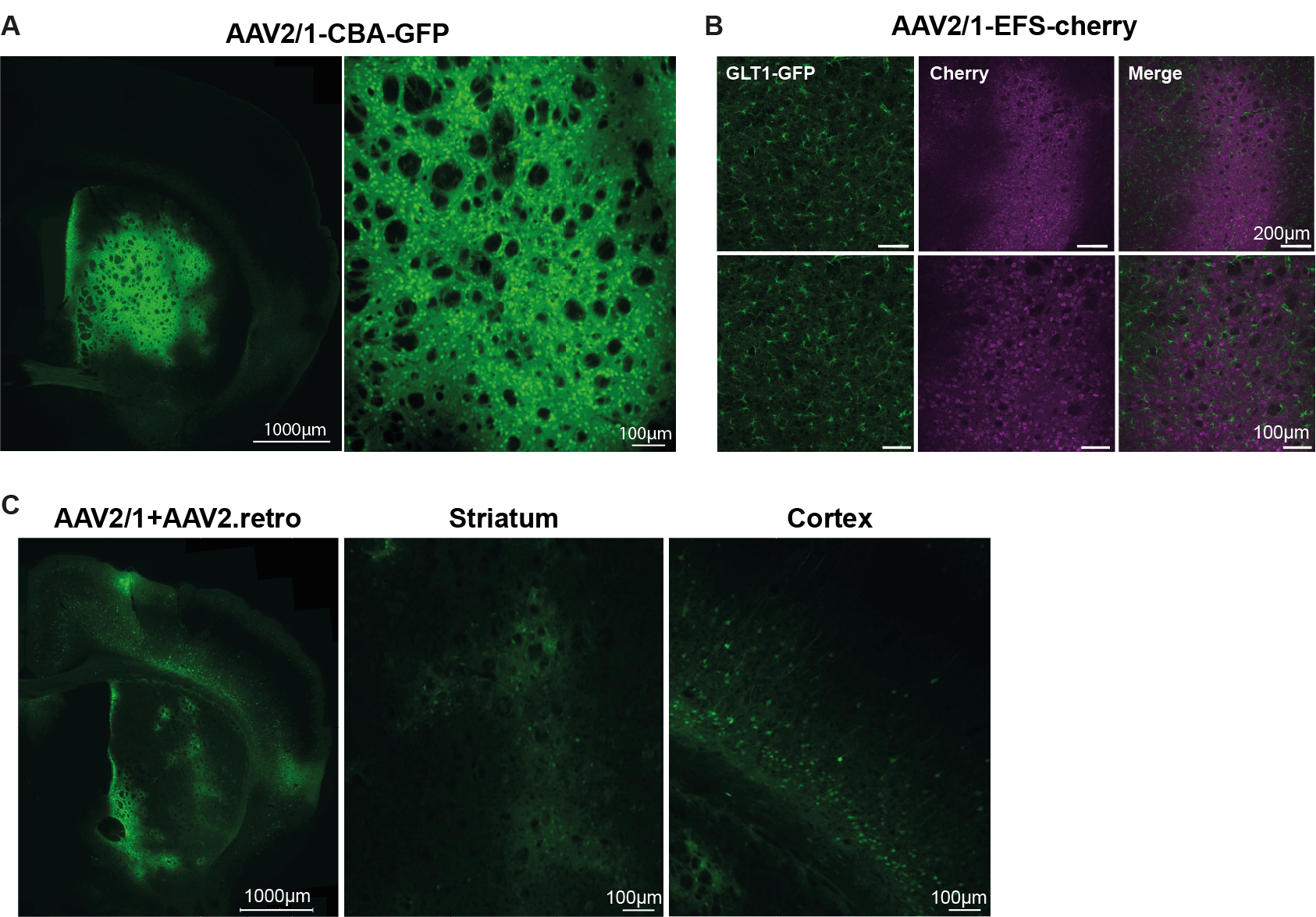
**

**Supplementary Figure 1: Transduction and retrograde properties of AAV2/1 and AAV2.retro expressing reporter genes encoding fluorescent proteins. (A)** Broad diffusion and transduction of striatal cells after the delivery of AAV2/1-CBA-GFP. **(B)** An AAV2/1 expressing mCherry under the control of the EFS promoter was injected into BAC-GLT1-GFP transgenic mice. These mice endogenously expressed GFP in astrocytes. The weak co-localization between signals suggests that AAV2/1-EFS-mCherry has a strong neuronal tropism, as previously reported for AAV2/rh10-EFS-cherry. [^1^](#_ENREF_1) **(C)** Co-injection into the striatum of AAV2/1 and AAV2.retro expressing GFP under the control of the CBA promoter led to poor transduction in both the cortex and striatum.

**
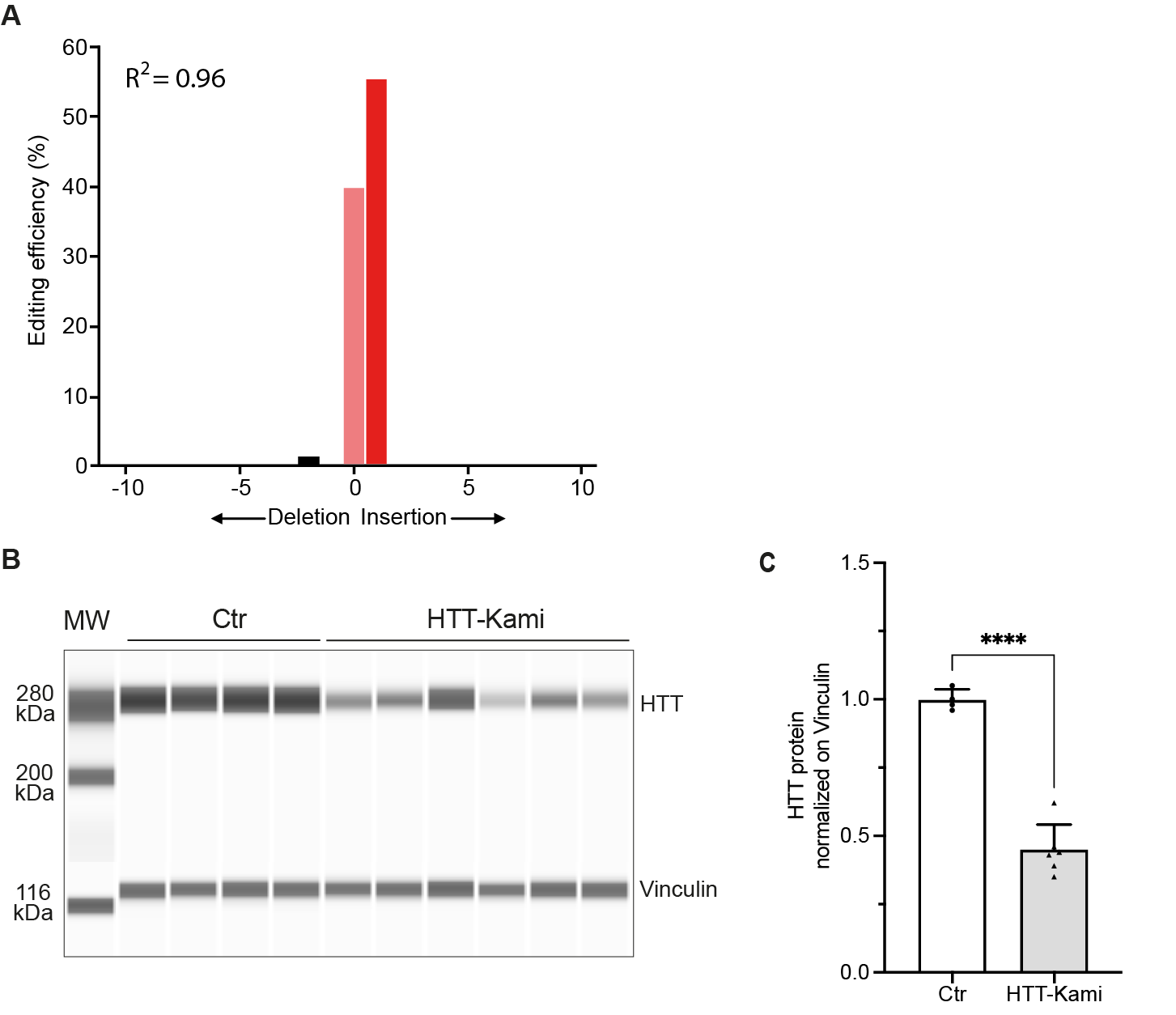
**

**Supplementary Figure 2: TIDE analysis and capillary-based immunoassay. (A)** TIDE analysis showing the editing pattern of sgHTT51 with most edited *mmHTT* alleles presenting the insertion of an adenine residue (+1A). **(B)** We confirmed that editing wild-type *HTT* with sgHTT51 resulted in gene inactivation, by analyzing HTT protein levels in a capillary western blot assay with the 4C8 antibody. **(C)** The vinculin antibody was used as an internal standard for the quantitative analysis (*n* = 4 punch specimens for the control and *n* = 6 punch specimens for the HTT-KamiCas9 group).

**
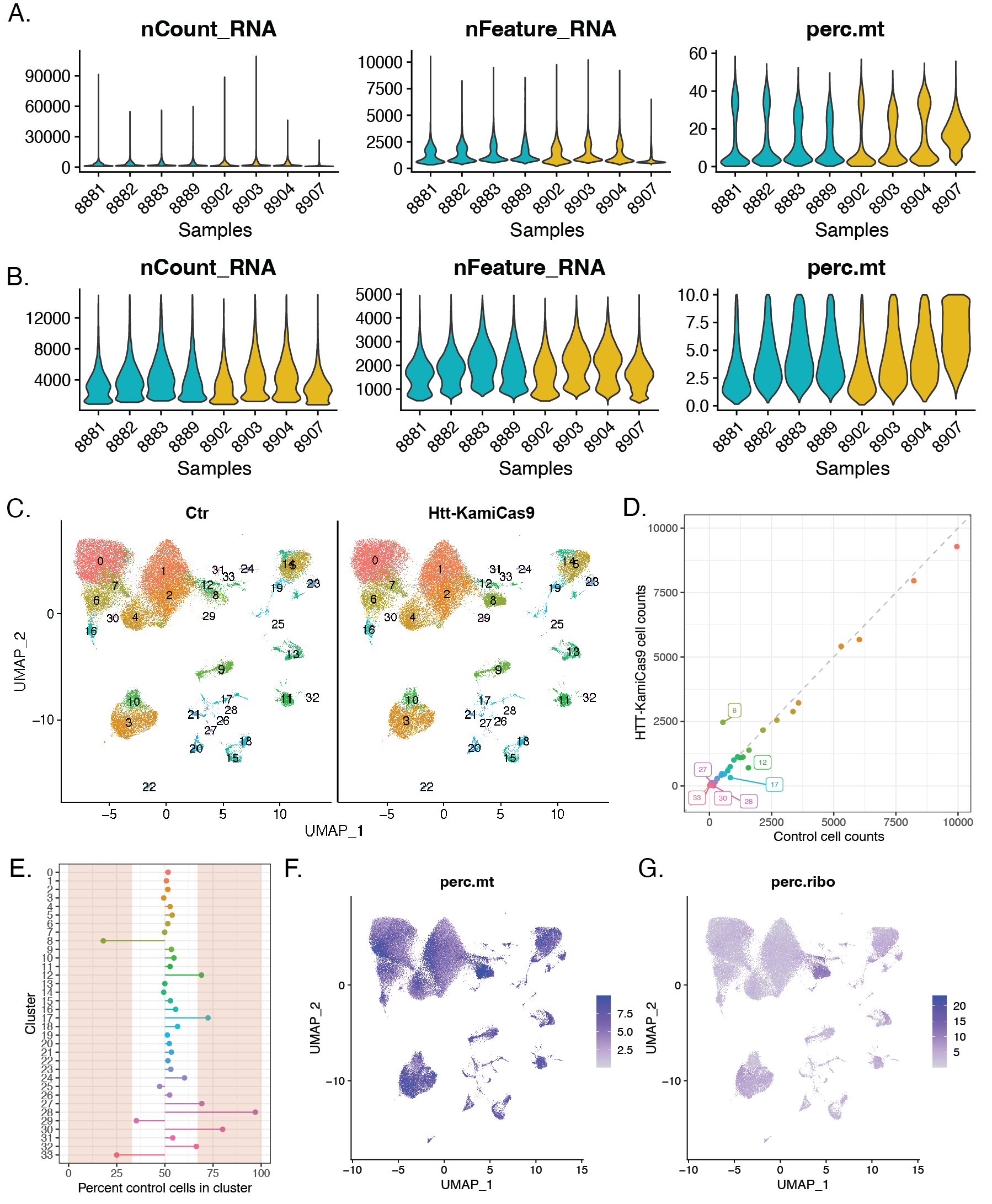
**

**Supplementary Figure 3: Single-nuclei RNA-seq quality control**. Single-nuclei statistics for the number of UMI counts, the number of genes and the percent mitochondrial expression before **(A)** and after **(B)** filtering steps. **(C)** UMAP of seurat-called clusters in both the control (left) and HTT-KamiCas9 (right) animals. **(D)** Dotplot indicating the number of cells in each cluster in the control (*x*-axis) and HTT-KamiCas9 (*y*-axis) animals. Clusters with a difference of more than 30% between treatments are labelled. **(E)** Lollipop plot indicating the percentage of cells in each treatment. UMAP colored according to percent mitochondrial reads **(F)** and percent ribosomal reads **(G)**, indicating that cluster 8 corresponds principally **to** cells with high levels of contamination. *n* = 4 biological replicates/group.

**
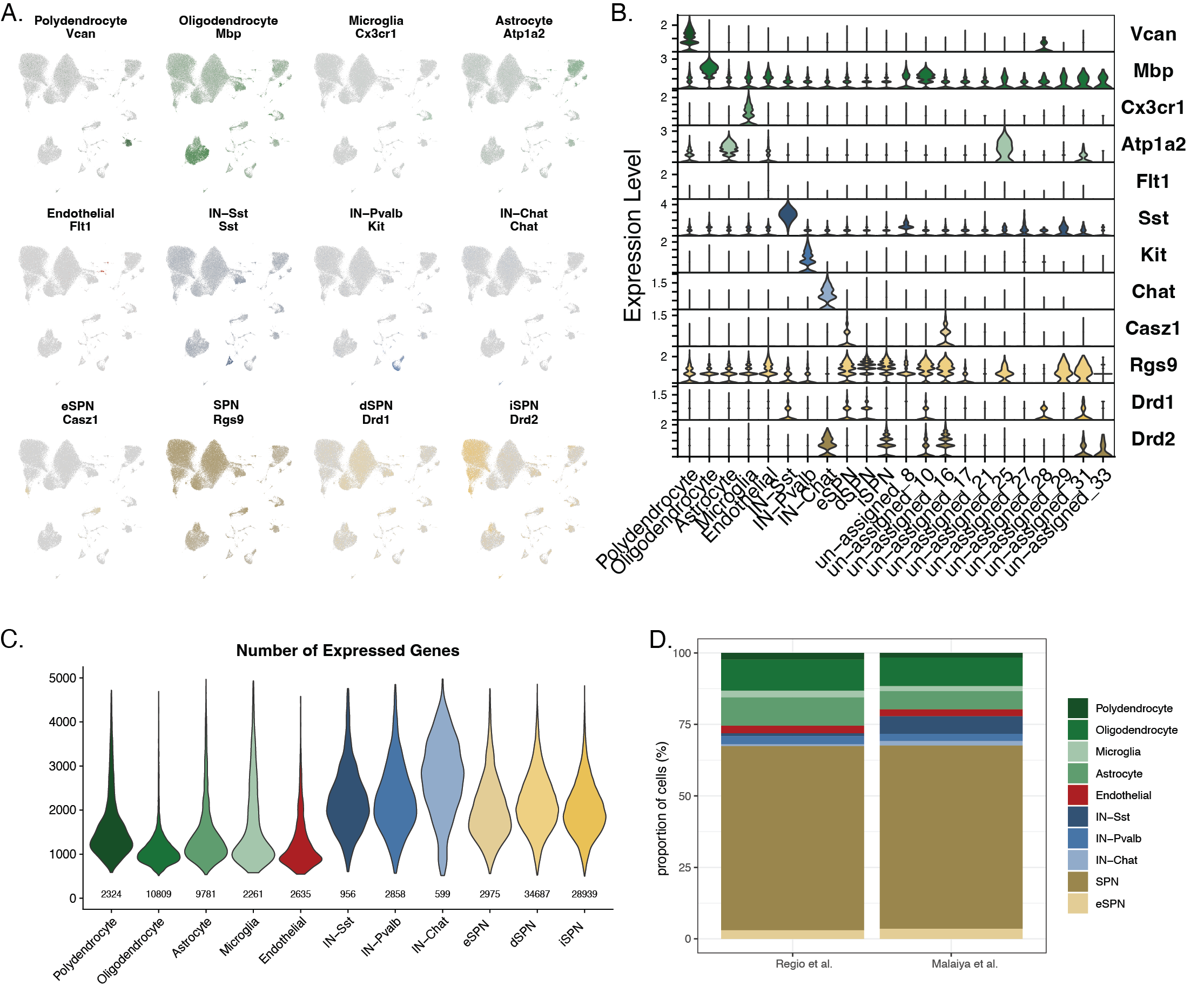
**

**Supplemental Figure 4: Single-nuclei RNA-seq cell-type determination.** **(A)** UMAP plots of cell-type markers for each of the indicated cell types (from Malaiya and coworkers). **(B)** Violin plots of cell-type marker expression in each cell type. **(C)** Number of genes expressed in each cell type. Neurons express a large number of genes than glia. The values below each violin represent the numbers of cells in that group. **(D)** The proportions of cells assigned to each cell type (left column) were similar to those reported by Malaiya and coworkers. *n* = 4 biological replicates/group.

**
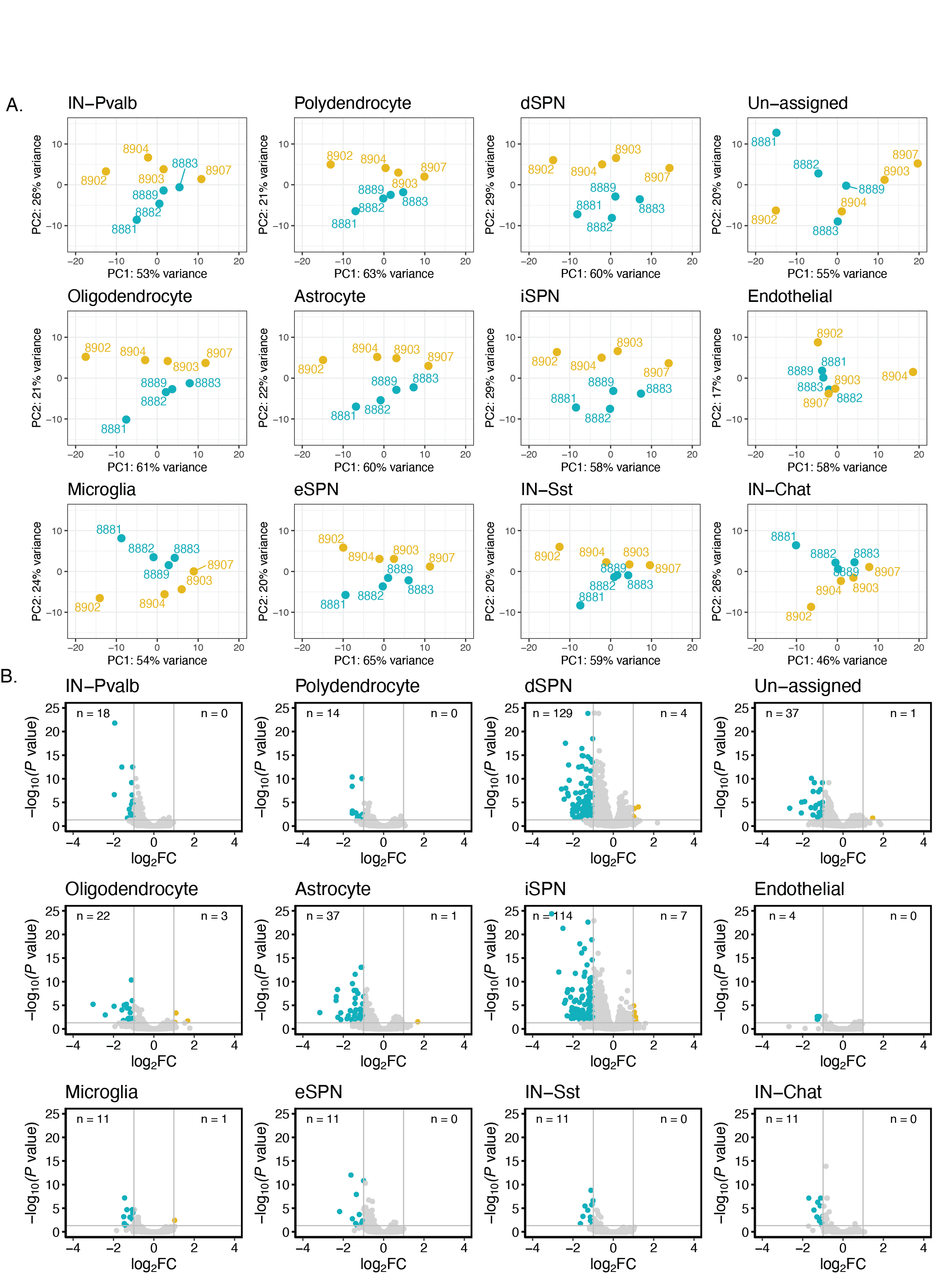
**

**Supplemental Figure 5: Single-nuclei RNA-seq differential expression**. **(A)** PCA plots based on the top 500 most variable genes in each cell type. Each point represents one library, color-coded by treatment (Ctr (blue) and HTT-KamiCas9 (yellow)). **(B)** Volcano plots showing the magnitude of differential expression (log_2_FC ≥ 1; adjusted *p*-value ≤ 0.05) versus statistical significance (-log_10_ *P*-value) between HTT-KamiCas9 samples and Ctr samples for each cell type. *n* = 4 biological replicates/group.

**Supplemental Figure 6:** Common downregulated genes in dSPN and iSPN (log2FoldChange < -1 &amp; p-adjusted< 0.05) shown as fraction of expressing cells (circle size) and mean expression (color) across defined cell types.

**
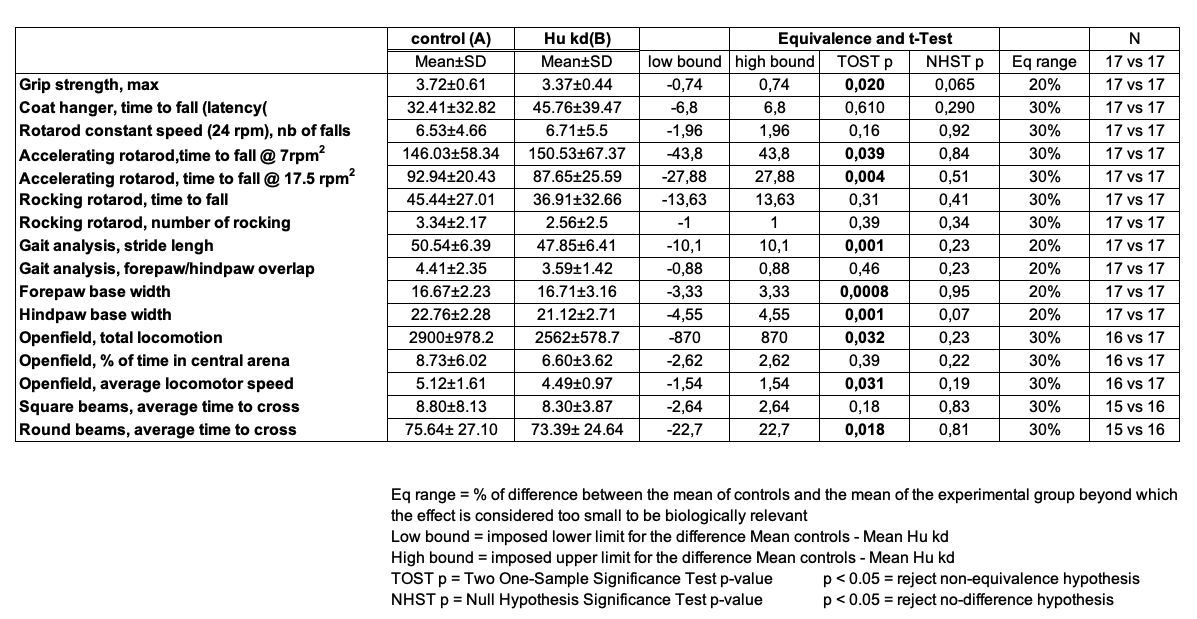
**

**Supplementary Table 1: Equivalence test for the behavioral analysis**

**Materials and Methods**

**Plasmids**

KamiCas9 lentiviral vectors (LV): For the development of an optimized LV-KamiCas9 system, the following plasmids were used: SIN-cPPT-PGK-spCas9-WPRE, SIN-cPPT-CMV-spCas9-V5-WPRE, SIN-cPPT-H1-sgHT1-PGK-mCherry-WPRE, SIN-cPPT-H1-sgHTT1-7SK-sgCas9_1-PGK-mCherry-WPRE ^[2](#_ENREF_2)^, SIN-cPPT-PGK-GFP-WPRE ^[3](#_ENREF_3)^.The second generation LV-CRISPR and LV-KamiCas9 were generated by replacing the H1 promoter and tracr sequence with the U6 promoter and optimized tracr ^[4](#_ENREF_4)^. A pMK-AttL1-U6-BsaI-optimized tracr-U6-SapI-optimized tracr-7sk-BsmBI tracr-AttL2 was ordered from Geneart (Invitrogen, Life Technologies, Zug, Switzerland). The tracrRNA was optimized with an extended loop (+5 bp) and a mutation of the fourth uracil of the sRNA polyT signal ^[4](#_ENREF_4)^. Type II restriction enzyme sites were inserted between the PolIII promoter and the tracrRNA to facilitate the “scarless” insertion of sgRNA sequences. The sgHTT1_F(SapI) ACCGACCCTGGAAAAGCTGATGA and sgHTT1_R(SapI) AACTCATCAGCTTTTCCAGG GTC oligomers were used to clone the guide sgHTT1 and to generate the pMK-AttL1-U6-BsaI-tracrRNA-U6-sgHTT1-7sk-BsmBI-tracrRNA-AttL2 plasmid. The U6-BsaI-optimized tracr and 7sk-BsmBI tracr cassettes were removed by digestion with BamHI and XbaI to generate pMK-U6-sgHTT1. For the KamiCas9 version, the U6-BsaI-tracrRNA cassette was removed by BamHI digestion to generate pMK-AttL1-U6-sgHTT1-7sk-BsmBI-tracrRNA-AttL2. The sgCas9_F(BsmBI) CCTCAATGGAGTACTTCTGGTCGA and sgHTT1_R(BsmBI) AAACTCGACCAGAAG TACTCCATT oligomers were used to clone the guide sgCas9 and to generate the pMK-AttL1-U6-sgHTT1-7sk-sgCas9_2-AttL2 plasmid. Finally, the guide RNAs were transferred to the LV destination vector SIN-cPPT-Gateway-PGK-mCherry-WPRE ^[2](#_ENREF_2)^ to produce the second-generation constitutive and KamiCas9 vectors: SIN-cPPT-U6-sgHTT1-7sk-sgCas9_1-PGK-mCherry-WPRE and (SIN-cPPT-U6-sgHTT1-PGK-mCherry-WPRE.

LV-mouse HTT reporter and mouse HTT sgRNA: For generation of the mouse reporter gene for *in vitro* sgRNA evaluations, the region expressing the first 171 aa of the mouse HTT gene, including part of the 5’UTR, was amplified from cDNA isolated from a C57BL/6 mouse with the following primers: HTT-7Q-5'UTR: CAC CGG ATC CGT GCC AGT AGG CTC CAA GTC TTC AGG GTC TGT CCC ATC GGG CAG GAA GCC GTC ATG; mHTT reporter_R: TTA AGC GTA ATC TGG AAC ATC GTA TGG GTA TCG AAC CAG GTG AGC CAG CTC. The PCR product was inserted into a pENTR/D-TOPO vector (Invitrogen, Life Technologies, Zug, Switzerland) and transferred into the destination vector SIN-cPPT-PGK-Gateway-WPRE ^[5](#_ENREF_5)^ to generate SIN-cPPT-PGK-mouse HTT171-7Q-WPRE. The forward and reverse oligomers sgHTT51_F(SapI): ACC**G**AACCCTGGAAAAGCTGATGA, sgHTT51_R(SapI): AACTCATCAGCTTTTCCAGGGTTC were used for cloning in the pMK-AttL1-U6-BsaI-tracrRNA-U6-SapI-tracrRNA-7sk-BsmBI-tracrRNA-AttL2 plasmid (Invitrogen, Life Technologies, Zug, Switzerland). The first U6 expression cassette and 7sk cassette were removed by digestion with BamHI and XbaI to generate pMK-AttL1-U6-sgHTT51-AttL2. An LR reaction was used to generate SIN-cPPT-U6-sgHTT51-WPRE. The optimized KamiCas9 version was generated with the sgCas9_1F(BsmbI): CCT CAA TGG AGT ACT TCT TGT CCA and sgCas9_1R(BsmbI): AAA CTG GAC AAG AAG TAC TCC ATT oligomers and pMK-AttL1-U6-sgHTT51-tracrRNA-7sk-BsmBI-tracrRNA-AttL2 digested with BsmbI. An LR reaction was then performed with SIN-cPPT-Gateway-WPRE to generate SIN-cPPT-U6-sgHTT51-7sk-sgCas9_1-WHV.

Constitutive AAV-SpCas9 plasmids:

The spCas9 nuclease was expressed from pX551-CMV-SpCas9 (A gift from Alex Hewitt RRID:Addgene_107024 ; pAAV2-CMV-HA-SV40nls-SpCas9-SV40nls-SYNpA_px551) or pAAV-EFS-SpCas9 (A gift from Ryohei Yasuda ; RRID:Addgene_104588 ; pAAV2-EFS-myc-SV40nls-spCas9-SV40nls-SYNpA) [^6^](#_ENREF_6). The kash sequence at the 3’ end of the GFP from the AAV transfer plasmid pX552-CMV-GFP (A gift from Alex Hewitt, RRID:Addgene 107023) was removed and a stop codon was added to generate pAAV2ss-U6-SapI-sgRNA-CMV-GFP-hGH. The sgHTT51 guide RNA targeting the mouse huntingtin gene (*HTT*) was generated with the forward and reverse primers sgHTT51_F(SapI): ACC**G**AACCCTGGAAAAGCTGATGA, sgHTT51_R(SapI): AACTCATCAGCTTTTCCAGGGTTC (Microsynth, Balgach, Switzerland) and hybridized to form double-stranded fragments with SapI overhangs. We inserted sgHTT51 into a SapI restriction site in pX552-CMV-GFP to generate pAAV2-U6-sgHTT51-CMV-GFP. We used pAAV2ss-CBA-AcGFPnuc-WPRE-bGH to identify transduced cells *in vivo*.

AAV-KamiCas9 plasmids: We generated pAAV2ss-EFS-GFP-SYNpA from pAAV-EFS-SpCas9 (a gift from Ryohei Yasuda ; RRID:Addgene_104588 ; pAAV2-EFS-myc-SV40nls-spCas9-SV40nls-SYNpA) ^[6](#_ENREF_6)^ by replacing spCas9 (AgeI/EcoRI) with the GFP reporter gene from SIN-cPPT-PGK-GFP-WPRE ^[3](#_ENREF_3)^. We designed a new sgCas9_2 for the AAV-KamiCas9 system, using two oligomers, sgCas9_2F(BsmBI): CCTCGTCGCCGAAGAAAAAGCGCA, sgCas9_2R(BsmBI): AAACTGCGCTTTTTCTTCGGCGAC, recognizing the myc-SV40nls sequence present at the 5' end of the spCas9 of the pAAV-EFS-SpCas9 vector. The pMK-AttL1-U6-sgHTT51-7sk-BsmBI-tracrRNA-AttL2 plasmid was digested with BsmbI for insertion of the sgCas9 and generation of the pMK-AttL1-U6-sgHTT51-7sk-sgCas9_2-AttL2 plasmid. This plasmid was then used to insert the optimized cassettes into the two AAV vectors, pAAV2ss-EFS-GFP-SYNpA-U6-sgHTT51-U6-sgCas9_2 and pAAV2ss-U6-sgHTT51-7sk-sgCas9_2-bGH.

**HEK 293T cell culture and transfection**

The day before transfection, 5x10^5^ cells were plated in six-well plates. The calcium-phosphate method was used for transfection. We mixed up to 4 μg of plasmids in 0.25 M CaCl_2_ solution and the mixture was then added dropwise to HEPES saline buffer (Sigma-Aldrich, Buchs, Switzerland, CaCl_2_-H_2_O:HEPES ratio 1:1). The mixture was incubated at room temperature for five minutes and added dropwise to the cells (10% of the culture volume). The medium was completely replaced 6 h after transfection.

The functionality of the *mmHTT r*eporter gene was assessed by transfecting HEK-293T cells with 3 μg of SIN-cPPT-PGK-5’UTR-mouse HTT171-7Q-HA-WPRE. As negative controls, cells were transfected with SIN-cPPT-PGK-HTT171-18Q-myc-WPRE ^[3](#_ENREF_3)^. Cells were fixed 72 hours after transfection for immunofluorescence analysis (Fig. 2E).

Mouse *HTT* editing was assessed by transfecting HEK-293T (Fig. 2) cells with 1 μg of the *mmHTT* reporter gene (SIN-cPPT-PGK-5’UTR-mouse HTT171-7Q-HA-WPRE), 1.5 μg of pAAV2-U6-sgHTT51-CMV-GFP and 1.5 μg of pAAV2-CMV-HA-SV40nls-SpCas9-SV40nls-SYNpA_px551. As a negative control, cells were transfected with pAAV2-CMV-HA-SV40nls-SpCas9-SV40nls-SYNpA_px551 and SIN-cPPT-PGK-GFP-WHV. All samples were lysed and analyzed 3 days post-transfection.

The expression of the mouse *HTT* reporter gene was checked by immunofluorescence analysis with the HA antibody (Fig. 2E-F). HEK-293T cells were transfected with 3 μg of SIN-cPPT-PGK-5’UTR-mouse HTT171-7Q-HA-WPRE, 0.5 μg of pAAV2-U6-sgHTT51-CMV-GFP and 0.5 μg of pAAV2-CMV-HA-SV40nls-SpCas9-SV40nls-SYNpA_px551 (*N*=1, *n*=2). All samples were analyzed 3 days post-transfection.

**Primary striatal cocultures and transduction**

Striatal neurons were infected on day 2 with 30 ng SIN-cPPT-PGK-SpCas9-WHV alone (control) or together with 30 ng SIN-cPPT-U6-sgHTT51-7sk-sgCas9-WHV (KamiCas9) and were then cultured for a further 12 days. Half the medium was replaced weekly. On day 12 post-infection, DNA was extracted with QuickExtract (Lubioscience, Zurich, Switzerland) and editing efficiency was analyzed by TIDE.

**Culture and transduction of neuronal precursor cells (NPCs)**

The day before transduction, NPCs were plated at a density of 1.5 x 10^6^ per well in six-well plates. NSCs were transduced with LV diluted in PBS-1%BSA (BSA, Sigma-Aldrich, Buchs, Switzerland, in a final volume of 15µL. Diluted LVs (100 ng p24 for each vector) were added directly to the medium. The medium was entirely replaced 12-24 h post-transduction. For optimization of the KamiCas9 system, we first compared vectors expressing Cas9 under the control of the PGK or CMV promoters (Fig. 1A-D).

NPCs were transduced with 100 ng p24 antigen from SIN-cPPT-PGK-Cas9-WPRE or SIN-cPPT-CMV-Cas9-WPRE and 100 ng p24 antigen from the constitutive SIN-cPPT-H1-sgHTT1-PGK-mCherry-WPRE vector or the KamCas9 vector SIN-cPPT-H1-sgHTT1-7sksgCas9_1-PGK-mCherry-WPRE. Negative controls were transduced with 100 ng p24 antigen from SIN-cPPT-PGK-Cas9-WHV. Cells were passaged seven days after transduction and lysed three weeks post-transduction. In a second experiment, the H1 and U6 promoters were evaluated (Fig. 1E-H). NPCs were transduced with 100 ng p24 antigen from SIN-cPPT-PGK-Cas9-WPRE and 100 ng p24 antigen from one of the constitutive vectors, SIN-cPPT-H1-sgHTT1-PGK-mCherry-WPRE or SIN-cPPT-U6-sgHTT1-PGK-mCherry-WPRE, or one of the KamiCas9 vectors, SIN-cPPT-H1-sgHTT1-7sksgCas9_1-PGK-mCherry-WPRE or SIN-cPPT-U6-sgHTT1-7sk-sgCas9_1-PGK-mCherry-WPRE. Negative controls were transduced with 100 ng p24 antigen from SIN-cPPT-PGK-Cas9-WHV and SIN-cPPT-PGK-GFP-WHV. Cells were passaged seven days after transduction and lysed two weeks post-transduction.

**Surgery**

Mice received a total volume of 4-7 µL of virus suspension per site, administered at a rate of 0.5 µL/min. AAV2/1 (8x10^8^ vg/hemisphere) (Fig. 3B, E) and AAV2/rh10 8x10^8^ vg/hemisphere) (Fig. 3D, G) were administered bilaterally at a single striatal site per hemisphere (coordinates 1 mm rostral to bregma; ±1.8 mm lateral to midline; and 3.5 mm ventral from the skull surface, with the tooth bar set at -3.3 mm). For AAV2.retro, two striatal sites were initially used (coordinates: +1.2 ; ±2.0 ; -3.2 (site 1) and +0.26 ; ±2.8 ; -3.2 (site 2) (4x10^8^ vg/hemisphere) (Fig. 3C, F). The needles were left in place for five minutes after the injection and were then slowly removed. The skin was sutured with 6-0 Prolene sutures (B-Braun Medical SA, Sempach, Switzerland) and healing cream was applied to the scalp (Bepanthen®Plus, Bayer, Leverkusen, Germany). Sterile 0.9% NaCl (B-Braun Medical SA, Sempach, Switzerland) was delivered by subcutaneous injection to prevent dehydration and the mice were allowed to recover on a heating mat. The mice received 2 mg/mL sweetened acetaminophen (Dafalgan®, UPSA, Agen, France) in water for three days following the intervention, for pain relief.

In the final experiment (Fig. 5), eight-week-old female mice of the FVB/N inbred strain were purchased from Janvier Laboratories, France. They were housed in groups in open-top type II L cages lined with aspen bedding, under standard conditions (light cycle of 12/12 h, temperature of 22°-24°). AAV2.retro and AAV2/Rh.10 (1.5x10^9^ vg/hemisphere) were injected at one site per hemisphere (*n*=18/group) (coordinates: +0.6; ±1.8; -3.2). Two mice (one in each group) displayed poor postsurgical recovery and were excluded from the study. Behavioral analysis was performed on the remaining animals (*n*=17/group). Immunofluorescence analysis was performed on 9.5-month-old animals (*n*=4/group). Editing efficiency was measured in the striatum and cortex of 11-month-old animals (*n*=6/group). Finally, single nuclei RNA sequencing was performed on striatal punches from 11.5-month-old animals (*n*=4/group). Four animals were excluded from the study because of poor transduction.

**Behavioral analysis**

Open-field tests

Open-field tests evaluating spontaneous locomotor activity, exploration, and rearing activity are among the most valuable tests for behavioral analyses. Mice were released individually in the center of a square arena (42 x 42 cm) under indirect, dim lighting (20 lux) and their locomotion was tracked by video for 10 minutes (EthoVision XT, Noldus, NL). The variables assessed were total distance covered, and the distance and time spent in the central zone of the arena.

Accelerating rotarod test

Accelerating rotarod tests were performed because this test reveals deficits in younger animals than constant-speed protocols ^[7](#_ENREF_7)^. Procedural motor learning, resistance to fatigue and motor coordination were assessed over five days in rotarod tests (Ugo Basile, Comerio, Italy). On day 1, the mice were acclimated to the test conditions such that they could remain for 180 seconds on a rod rotating at a constant speed of 18 rpm. On day 2, mice were tested for their ability to maintain gait for 300 seconds with the rod turning at a constant speed of 24 rpm, and the number of trials required to achieve this feat was recorded. On days 3 and 4, mice were tested for coordination and balance in two trials per day with a rod rotating at speeds accelerating from 5 to 40 rpm over 5 minutes and 2 minutes, respectively. The variable measured was the mean time to fall, or latency. On day 5, mice were subjected to two trials on the rod, which initially turned at a speed of 5 rpm, subsequently accelerating at a rate of 8 rpm/minute, with an inversion of the direction of rotation every 12 seconds. The variables measured were the time to fall and the number of rocking events (changes of direction).

Grip strength

Grip strength is affected in many mouse models of HD ^[8](#_ENREF_8)^. Mice handled by the tail were allowed to use their forepaws to grip a wire mesh connected to a digital dynamometer (grip strength meter, Columbus Instruments, Ohio, USA), and were then gently pulled by the tail until they released their grip. The test was repeated three times, in rapid succession, and the best value was recorded.

Coat hanger test

Mice were rapidly lifted by the tail and allowed to use their forepaws to grip the middle of a 60 cm-long metal bar (2.5 mm) suspended on two 20 cm-high escape ramps. The variables assessed were the time taken to reach one of the escape ramps with all four paws or, in the case of a fall, the time to the fall. Data for both variables were censored at 120 seconds.

Beam walking

Balance was assessed by determining the ability of the animal to reach a familiar shelter from an elevated platform by crossing a beam, in consecutive tests with a series of 100 cm-long beams of decreasing width or cross section, as previously described ^[9](#_ENREF_9)^. Padded diapers were placed on the floor, 40 to 50 cm below the beam, to absorb urine and prevent injury in case of fall. The mice were first trained to run this homing trail in sessions in which they were allowed to reach the shelter on the largest, flat beam by covering distances increasing from 20 to 80 cm. We used five square beams with widths of 24, 18, 12, 9 and 6 mm, followed by five round, slightly sanded aluminum beams of 16, 12, 9, 6 and 4 mm in diameter.

The mice underwent two consecutive trials on each beam, and the shortest time was recorded for each beam. The variable measured was the time taken to cross the central 80 cm of the beam.

Gait analysis

Gait abnormalities have also been observed in many mouse models of HD ^[8](#_ENREF_8),[10-12](#_ENREF_10)^. Gait was analyzed in a similar setting to that used for the beam-walking test, but with a Perspex runway measuring 50 x 8 x 8 cm, lined with paper. Red and black water-based endorsing ink (Tatone, China) was applied to the fore- and hindpaws, respectively, such that the mice led a trail of footprints along their passage. Stride length, overlap between fore- and hindpaw prints, and distance between left and right forepaws and hindpaws (i.e. the forepaw/hindpaw base) were averaged over 3 to 6 clear successive steps.

**TIDE analysis**

The following primers were used to measure editing efficiency: human HTT: TTGCTGTGTGAGGCAGAACCTGCGG and TGCAGCGGCTCCTCAGCCAC (Fig. 1), mouse HTT: CCTCCTCACTTCTTTTCTATCG and AGCATTATGTCATCCACTACC (Fig. 2), mouse HTT reporter gene: CTTCAAAAGCGCACGTCTGC and AGCTCAGCAAACCTCCACAG (Fig. 2), LV-PGK-spCas9: AATGGAAGTAGCACGTCTCACTAG and AGC GCG AGA TAG ATC AAC CG (Fig. 1), LV-CMV-spCas9: GTGTACGGTGGGAGGTCTATATAAG and AGC GCG AGA TAG ATC AAC CG (Fig. 2). For each PCR, we included a negative control in which the gDNA was replaced with sterile water (Gibco, Life Technologies, Zug, Switzerland). If the amplification yield was too low to obtain the amount of PCR product required for the TIDE analysis, multiple PCRs were performed, and their products were pooled before the purification step. PCR products were subjected to electrophoresis in an agarose gel and purified with the Nucleospin Gel and PCR clean-up kit (Macherey-Nagel, Oensingen, Switzerland) according to the manufacturer’s protocol. The concentration of the PCR product was determined with a Nanodrop spectrophotometer, and the PCR product was sequenced for each sample (Microsynth, Balgach, Switzerland).

**Off-target analysis**

Potential off-target sites were assessed with the Bioconductor package CRISPRseek [^13^](#_ENREF_13) with a maximum mismatch of 4. Sites with a score ≥1 were considered to be potential off-target sites.

**Capillary-based western blotting**

The primary anti-huntingtin antibody clone 1HU-4C8 (MAB2166, Zug, Switzerland) (diluted 1/500) and the anti-vinculin antibody clone 3M13 (ZRB1089, Merck, Nottingham, UK) (diluted 1/50) were used to target the HTT and vinculin proteins, respectively. HTT and vinculin were then detected by chemiluminescence with anti-rabbit-HRP conjugate (043-026, ProteinSimple, Bio-Techne AG, Zug Switzerland) diluted 1/20 in ready-to-use anti-mouse-HRP conjugate (DM-002, ProteinSimple, Bio-Techne AG, Zug, Switzerland). Compass software (version 6.1) was used for the analysis. Peaks were determined with the dropped-line method and the HTT and vinculin signals were used to calculate HTT/vinculin ratios.

**Immunofluorescence analysis**

For the immunofluorescence labeling of brain sections, free-floating coronal sections (25 µm) were washed three times, for 10 minutes each, in 1 x TBS-T (15 mM NaCl, 0.1% Triton X-100 10 mM Tris pH 7.6) and blocked by incubation for 1 h in 1 x TBS-T supplemented with 5% normal goat serum (NGS for the D7F7 antibody), 10% NGS (GFAP antibody), 10% BSA (IbaI antibody). Primary antibodies were diluted in 1 x TBS-T, 1% BSA (D7F7, IbaI) 10% NGS (GFAP) and incubated overnight with the sections at 4°C. The following primary antibodies were used: mouse monoclonal anti-HA antibody (RRID: AB_291262; HA.11 clone 16B12 cat#MMS-101R; dilution 1/1000; Biolegend Europe BV, Amsterdam, the Netherlands) (Fig. 2E), goat polyclonal anti-cherry antibody (RRID: AB_2333093, dilution: 1/250, Sicgen, Carcavelos, Portugal) (Fig. S1B), monoclonal rabbit anti-HTT D7F7 antibody (RRID: AB_10827977; dilution 1/500, **BioConcept,** Allschwil, Switzerland) (Figure 5D), mouse monoclonal anti-GFAP-Cy3 antibody (RRID: AB_476889; dilution 1/800, Sigma-Aldrich, Buchs, Switzerland) (Figure 5G), rabbit polyclonal anti-DARPP-32 antibody (RRID: AB_10807019, dilution: 1/500, Sigma-Aldrich Ab10518, Buchs, Switzerland) (Figure 5G), goat polyclonal anti-IbaI (RRID: AB_2224402, dilution: 1/500, Abcam, Amsterdam, the Netherlands) (Figure 5G). The following day, D7F7 and Cherry slices were washed three times, for 10 minutes each, with 1 x TBS-T and then incubated for 1 h at room temperature with a goat anti-rabbit IgG AlexaFluor® 594 (dilution: 1/1000, Invitrogen, Life Technologies, Zug, Switzerland) or donkey anti-goat IgG Alexa Fluor® 568 (RRID: AB_142581, dilution: 1/1000, Life Technologies, Zug, Switzerland) in 1 x TBS-T, 1% BSA. IbaI slices were washed three times, for 10 minutes each, with 1 x TBS-T and then incubated for 1 h at room temperature with a biotinylated donkey anti-goat IgG (RRID: AB_2340396, dilution 1/1000, Milan Analytica, Rheinfelden, Switzerland) in 1 x TBS-T, 1% BSA. Slices were washed three times, for 10 minutes each, with 1 x TBS-T and then incubated for 1 h at room temperature in streptavidin-Cy3 (RRID: AB_2337244, dilution 1/800, Milan Analytica, Rheinfelden, Switzerland) in TBS-T. Slices were then washed three times, for 10 minutes each, in 1 x TBS-T and mounted on Superfrost+ slides, in Vectashield Fluorescence mounting medium supplemented with DAPI (Reactolab, Servion, Switzerland).

**scRNA-seq analysis with Seurat**

Filtered feature-barcode matrices corresponding to 291,920 cells across four replicates for treatment and control samples were loaded into R (v4.2.2) [^14^](#_ENREF_14) and analyzed with the Seurat (v4.2) package Hao [^15^](#_ENREF_15). The Seurat object was created with the command CreateSeuratObject(min.cells = 10), which requires genes to be expressed in at least 10 cells. Cells were filtered out if they expressed fewer than 300 or more than 5000 genes, had fewer than 500 or more than 15,000 UMI counts, or if they had over 10% mitochondrial counts, leaving 108,550 cells for analysis. The data were normalized by the SCTransform method, with the following parameters: method = “glmGamPoi”, vars.to.regress = “percent.mitochondria”, variable.features.n = 3000.

For cell clustering, we identified the top 50 PCs in principal component analysis (PCA). We identified 34 clusters with the commands FindNeighbors(dims = 1:50) and FindClusters(). UMAP dimensional reduction was performed with the Seurat function RunUMAP. Each cluster was assigned a cell type based on the expression of known cell-type markers (polydendrocytes: Pdgfra, Vcan; oligodendrocytes: Mbp, Plp1; microglia: Cx3cr1, C1qa; astrocytes: Slc1a2, Atp1a2; endothelial cells: Flt1, Rgs5; inhibitory-Sst: Npy, Sst; inhibitory-Pvalb: Kit, Nxph1; inhibitory-Chat: Chat, Ecel1; eSPN: Casz1, Otof; Ntng1; SPN: Rgs9, Pde10a; dopamine subtypes of dSPN and iSPN: Drd1, Drd2) ^[16](#_ENREF_16)^.

Augur ^[17](#_ENREF_17)^, a tool prioritizing the responsiveness of the population to experimental perturbations, was used with default parameters to calculate perturbation prioritization for each cell type and between the control and HTT-KamiCas9 samples.

Differential expression analysis was performed for each cell-type cluster separately, with DESeq2 (v1.36) [^18^](#_ENREF_18). Gene counts were first aggregated by adding together the counts of cells corresponding to the same library. Genes with fewer than 10 counts in at least four samples were removed. DESeq was run with the Wald test and a local fit type. Pairwise comparisons were then made by setting the contrast between knockdown and control samples. For downstream analysis, genes were considered to be differentially expressed if the absolute value of log_2_ fold change was greater than 1 (2-fold differences) and the adjusted *p*-value was less than 0.05.

**lncRNA analysis**

Analysis of repeats

Content and repeat numbers were analyzed with RepeatMasker (v4.1.5) for genes identified as differentially downregulated on scRNA-seq (log2FoldChange < -1 & *p*-adjusted < 0.05). [^19^](#_ENREF_19) We selected the longest isoform of each gene with GRCm39 GENCODE annotation (v27). [^20^](#_ENREF_20) We then investigated the percentage of the sequence covered by repeats, its chromosomal frequency and the distribution of repeat classes within the selected genes. Finally, we compared this distribution of repeat classes to that for all the lncRNAs annotated in the GRCm39 GENCODE annotation (v27).

Cell-type specificity analysis

In the scRNA-seq dataset, genes differentially downregulated (log_2_ fold change < -1 & *p*-adjusted < 0.05) in dSPN and iSPN relative to other cell types were identified and their cell-type specificity was investigated. Their normalized expression was represented with the DotPlot function of Seurat. [^21^](#_ENREF_21)

**REFERENCES**

1. Duarte F, Ramosaj M, Hasanovic E*, et al*. Semi-automated workflows to quantify AAV transduction in various brain areas and predict gene editing outcome for neurological disorders. *Molecular therapy Methods & clinical development*. Jun 8 2023;29:254-70.

2. Merienne N, Vachey G, de Longprez L*, et al*. The Self-Inactivating KamiCas9 System for the Editing of CNS Disease Genes. *Cell Rep*. Sep 19 2017;20(12):2980-91.

3. Pereira de Almeida L, Ross CA, Zala D, Aebischer P, Déglon N. Lentiviral-mediated delivery of mutant huntingtin in the striatum of rats induces a selective neuropathology modulated by polyglutamine repeat size, huntingtin expression levels, and protein length. *J Neurosci*. May 1 2002;22(9):3473-83.

4. Dang Y, Jia G, Choi J*, et al*. Optimizing sgRNA structure to improve CRISPR-Cas9 knockout efficiency. *Genome Biol*. Dec 15 2015;16:280.

5. Drouet V, Perrin V, Hassig R*, et al*. Sustained effects of nonallele-specific Huntingtin silencing. *Ann Neurol*. Mar 2009;65(3):276-85.

6. Nishiyama J, Mikuni T, Yasuda R. Virus-Mediated Genome Editing via Homology-Directed Repair in Mitotic and Postmitotic Cells in Mammalian Brain. *Neuron*. Nov 15 2017;96(4):755-68 e5.

7. Pallier PN, Drew CJ, Morton AJ. The detection and measurement of locomotor deficits in a transgenic mouse model of Huntington's disease are task- and protocol-dependent: influence of non-motor factors on locomotor function. *Brain Res Bull*. Mar 30 2009;78(6):347-55.

8. Menalled L, El-Khodor BF, Patry M*, et al*. Systematic behavioral evaluation of Huntington's disease transgenic and knock-in mouse models. *Neurobiol Dis*. Sep 2009;35(3):319-36.

9. Dunnett SB, Brooks SP. Motor Assessment in Huntington's Disease Mice. *Methods Mol Biol*. 2018;1780:121-41.

10. Carter RJ, Lione LA, Humby T*, et al*. Characterization of progressive motor deficits in mice transgenic for the human Huntington's disease mutation. *J Neurosci*. Apr 15 1999;19(8):3248-57.

11. Heng MY, Tallaksen-Greene SJ, Detloff PJ, Albin RL. Longitudinal evaluation of the Hdh(CAG)150 knock-in murine model of Huntington's disease. *J Neurosci*. Aug 22 2007;27(34):8989-98.

12. Menalled LB, Sison JD, Dragatsis I, Zeitlin S, Chesselet MF. Time course of early motor and neuropathological anomalies in a knock-in mouse model of Huntington's disease with 140 CAG repeats. *J Comp Neurol*. Oct 06 2003;465(1):11-26.

13. Zhu LJ, Holmes BR, Aronin N, Brodsky MH. CRISPRseek: a bioconductor package to identify target-specific guide RNAs for CRISPR-Cas9 genome-editing systems. *PLoS One*. 2014;9(9):e108424.

14. Team RC. R: A Language and Environment for Statistical Computing. <https://www.R-project.org/>

15. Hao Y, Hao S, Andersen-Nissen E*, et al*. Integrated analysis of multimodal single-cell data. *Cell*. Jun 24 2021;184(13):3573-87 e29.

16. Malaiya S, Cortes-Gutierrez M, Herb BR*, et al*. Single-Nucleus RNA-Seq Reveals Dysregulation of Striatal Cell Identity Due to Huntington's Disease Mutations. *J Neurosci*. Jun 23 2021;41(25):5534-52.

17. Squair JW, Skinnider MA, Gautier M, Foster LJ, Courtine G. Prioritization of cell types responsive to biological perturbations in single-cell data with Augur. *Nat Protoc*. Aug 2021;16(8):3836-73.

18. Love MI, Huber W, Anders S. Moderated estimation of fold change and dispersion for RNA-seq data with DESeq2. *Genome Biol*. 2014;15(12):550.

19. Smit AFA, Hubley R, Green P. RepeatMasker Open-3.0. <http://www.repeatmasker.org>

20. Frankish A, Diekhans M, Ferreira AM*, et al*. GENCODE reference annotation for the human and mouse genomes. *Nucleic Acids Res*. Jan 8 2019;47(D1):D766-D73.

21. Butler A, Hoffman P, Smibert P, Papalexi E, Satija R. Integrating single-cell transcriptomic data across different conditions, technologies, and species. *Nat Biotechnol*. Jun 2018;36(5):411-20.
